# High-abundance peaks and peak clusters associate with pharmaceutical polymers and excipients in urinary untargeted clinical metabolomics data: exploration of their origin and possible impact on label-free quantification†

**DOI:** 10.1039/d3an01874a

**Published:** 2024-01-03

**Authors:** Frank Klont, Fleur B. Nijdam, Stephan J. L. Bakker, Pekka Keski-Rahkonen, Gérard Hopfgartner, TransplantLines Investigators

**Affiliations:** a Unit of PharmacoTherapy, -Epidemiology & -Economics, Groningen Research Institute of Pharmacy, University of Groningen Antonius Deusinglaan 1 9713 AV Groningen The Netherlands frank.klont@rug.nl; b Department of Clinical Pharmacy and Pharmacology, University Medical Center Groningen, University of Groningen Hanzeplein 1 9700 RB Groningen The Netherlands; c Life Sciences Mass Spectrometry, Department of Inorganic and Analytical Chemistry, University of Geneva Quai Ernest Ansermet 24 1211 Geneva Switzerland; d Department of Internal Medicine, Division of Nephrology, University Medical Center Groningen, University of Groningen Hanzeplein 1 9700 RB Groningen The Netherlands; e Nutrition and Metabolism Branch, International Agency for Research on Cancer (IARC/WHO) Avenue Tony Garnier 25 69007 Lyon France; f Group of Authors on Behalf of the Transplant Lines Biobank and Cohort Study, University Medical Center Groningen, University of Groningen Hanzeplein 1 9700 RB Groningen The Netherlands

## Abstract

Pharmaceutical polymers and excipients represent interesting but often overlooked chemical classes in clinical exposure and bioanalytical research. These chemicals may cause hypersensitivity reactions, they can be useful to confirm exposure to pharmaceuticals, and they may pose bioanalytical challenges, including ion suppression in liquid chromatography-mass spectrometry (LC-MS-)based workflows. In this work, we assessed these chemicals in light of a rather surprising finding presented in two previously published studies, namely that usage of cyclosporine A, an immunosuppressive drug which is known to be cleared through excretion in the bile, explained the largest amount of variance in principal component analysis of urinary LC-SWATH/MS small-molecule profiling data. Specifically, we examined the freely-accessible 24-hour urine metabolomics data of 570 kidney transplant recipients included in the TransplantLines Biobank and Cohort Study (NCT03272841). These data unveiled thousands of high-abundance polymer peaks in some samples, which were associated with the use of the macrogol (*i.e.*, polyethylene glycol) 3350 oral laxative agent. In addition, we found multiple clusters of high-abundance peaks which were linked to the exposure to two pharmaceutical excipients, namely short-chain polyethylene glycol (molecular weight <1000 Da) and polyethoxylated castor oil (also known as Kolliphor® EL or Cremophor® EL). Respectively, these excipients are used in temazepam capsules and cyclosporine A capsules, and the latter provides a plausible explanation for the rather surprising finding that instigated our work. Moreover, such explanation and our findings in general put emphasis on taking into consideration these and other pharmaceutical polymers and excipients when exploring, processing, and interpreting clinical small-molecule profiling data.

## Abstract 2

Pharmaceutical polymers and excipients represent interesting but often overlooked chemical classes in clinical exposure and bioanalytical research.

## Graphical abstract

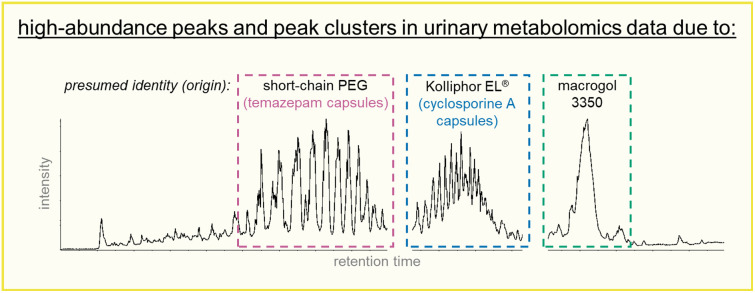

## 1 Introduction

Untargeted metabolomics by liquid chromatography coupled to mass spectrometry (LC-MS) is gaining momentum in clinical research, for example for identification of disease-related mechanisms, biomarkers, and druggable targets.^1,2^ This technique is furthermore used increasingly to obtain molecular evidence of chemical exposures, including dietary (*e.g.*, caffeine, ethanol), lifestyle (*e.g.*, tobacco smoke, illicit drugs), and medical exposures (*e.g.*, therapeutic drugs).^3–6^ Such evidence could enable the verification of clinical data retrieved through anamnesis and questionnaires, thus reducing various sources of bias in clinical exposure research.^4^ In addition, metabolomics can provide insights into un- and/or underreported exposures and into the real-world metabolism of xenobiotics, all representing rather underexplored application areas with considerably high clinical potential.^5,6^

For most applications of untargeted metabolomics, researchers can benefit from both the qualitative and quantitative opportunities offered by the corresponding techniques.^7,8^ However, there are still ample challenges relating to these two opportunities preventing metabolomics from achieving its full potential in clinical research. Many of these challenges are and can be tackled through development and application of advanced bioinformatics approaches.^9–11^ Nonetheless, the skills and critical eye of metabolomics researchers will likely remain crucial for enhancing data utilization, creating innovative applications, and finding novel areas of attention and improvement.

Regarding potential areas of attention, two recently published clinical metabolomics studies reported a similar and rather surprising finding which, we believe, warrants further investigation. The corresponding publications presented urinary metabolomics data of 688 ^5^ and 570 ^6^ stable kidney transplant recipients, and both papers featured principal component analysis (PCA) scores plots in which the largest amount of variance seemed to be explained by usage of the immunosuppressive drug cyclosporine A (CsA). This drug is, however, known to be excreted in the bile with only small percentages of the drug ending up in urine.^12^ CsA and its metabolites may thus be expected in urine, yet it is arguably unlikely that analytical signals derived from this exogenous compound explained more variance in PCA than, for example, usage of the drug mycophenolate (mofetil) which could be identified in the majority of urine samples in both studies,^5,6^ is given in much higher dosages (*i.e.*, grams rather than milligrams), and is known to be predominantly excreted in urine.

In this work, we aimed to provide a possible explanation for this unexpected observation for which we used one of the abovementioned datasets which are available through an open-access online data repository. In addition, we aimed to assess the corresponding findings in the context of the quantitative opportunities of the employed metabolomics workflow, with a distinct focus on the effectiveness of commonly used data normalization strategies, which aim to reduce the impact of various sources of unwanted (technical) variation.

## 2 Experimental

### 2.1 Clinical and metabolomics data

This study used existing 24-hour urine metabolomics data of 570 kidney transplant recipients included in the TransplantLines Biobank and Cohort Study (NCT identifier NCT03272841), which was approved by the Institutional Review Board of the University Medical Center Groningen (UMCG; decision METc 2014/077), which adheres to the UMCG Biobank Regulation, the Declaration of Helsinki, and the Declaration of Istanbul, and for which informed consent was obtained from all participants.^13^ The corresponding analyses have been described in detail elsewhere,^6^ and the metabolomics data have been deposited in an open-access data repository as can be found at: https://doi.org/10/gqmqtw (as sub-study 2).

### 2.2 Data analysis

Visual exploration of chromatograms and mass spectra was performed using SCIEX PeakView (version 2.2.0.11391), and all feature-based analyses were performed using a custom-modified version of SCIEX MarkerView (version 1.3.1) after first converting raw MS data to a list of peak area-based signal intensities for the aligned two-dimensional features (*i.e.*, mass-to-charge ratio (*m*/*z*), retention time) across the different samples using the same software. Regarding the feature data, these were either assessed directly or after applying most likely ratio-based normalization,^14^ total area sums-based normalization, median peak ratios-based normalization, normalization based on all five included internal standards, and normalization based on the diclofenac-^13^C_6_ internal standard only. See Table S1 in the ESI† for a detailed overview of data (pre)processing settings.

## 3 Results and discussion

### 3.1 Detection of pharmaceutical polymers and excipients

**Fig. 1:**
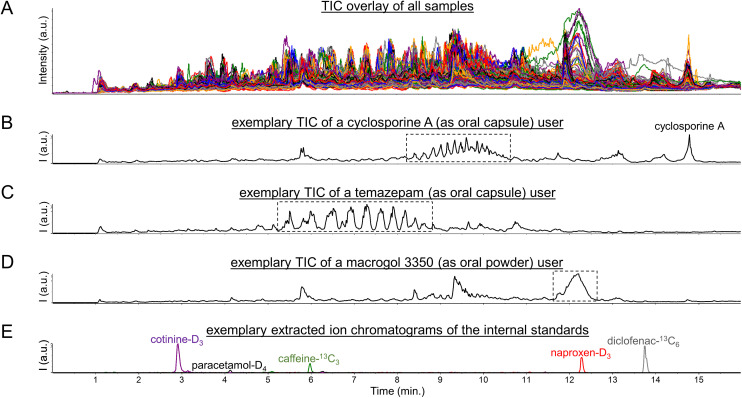
Total ion current chromatograms (TIC) of (A) all 570 urine samples, (B) a user of the immunosuppressive drug cyclosporine A, (C) a user of the short-acting benzodiazepine drug temazepam, and (D) a user of the laxative agent macrogol 3350, as well as (E) MS1 extracted ion chromatograms of the internal standards which were all added at 1 pmol (0.15–0.30 ng) per μL urine corresponding to 20 pmol (3–6 ng) injected onto the LC column. The Fig. S1, S3, and S6† furthermore include MS1 spectra of the peaks/regions highlighted with dotted-line boxes in the Fig. 1B, C, and D.

Initial exploration of the metabolomics data consisted of a visual inspection of the total ion current chromatograms (TIC), as are shown in Fig. 1A, which yielded several interesting findings. Firstly, CsA users could readily be spotted by solely looking at their TICs based on a large peak at 14.5 min reflecting CsA, as was described previously,^5^ and possible CsA metabolites (see Fig. 1B). Moreover, CsA users could also be spotted based on unusual clusters of peaks between 8 and 11 min (see Fig. 1B).

Regarding these clusters, corresponding mass spectra revealed 7 *m*/*z* differences between the doubly-charged peaks within each cluster (see Fig. S1 in the ESI†). In addition, 22 *m*/*z* differences were observed between the doubly-charged peaks in adjacent clusters (see Fig. S1 in the ESI†). These signals presumably reflect lipids with different numbers of methylene units (+14 Da) and different numbers of ethoxy groups (+44 Da), as we based on the fact that CsA is typically administered in an oral formulation containing polyethoxylated castor oil, also known as Kolliphor® EL or Cremophor® EL.^15^ When subsequently inspecting the principal component analysis (PCA) loadings plot of the metabolomics dataset (see Fig. S2 in the ESI†), many of these peaks were found as strong contributors to PC1. Thereby, we believe that this pharmaceutical excipient may have contributed to the previously reported observations^5,6^ that the first principal component in PCA analysis showed separation based on confirmed CsA use when analyzing urinary metabolomics data of kidney transplant recipients. However, the intensity levels of signals in the peak clusters between 8 and 11 min do not allow for a complete separation of CsA users from CsA nonusers (see Fig. S3 in the ESI†), as in some users these signals gave intensity values comparable to the background peaks in the corresponding regions. In this regard, it should be taken into account that the analyzed data originates from a study of renally-impaired individuals, hence urine might not be the most suitable biological matrix to determine drug exposure statuses.

A pattern of multiple high-abundance peaks comparable to those observed between 8 and 11 min was also seen in some study participants between 5 and 9 min (see Fig. 1c). The corresponding mass spectra (see Fig. S4 in the ESI†) did not show series of doubly-charged peaks with 7 and 22 *m*/*z* differences but rather series of singly-charged peaks with 44 *m*/*z* differences. These peaks could be identified by spectral library matching as ammonium adducts of short-chain (7- to 12-mer) polyethylene glycol molecules (see Fig. S5 in the ESI†). The presence of these peaks furthermore aligned reasonably well with usage of the short-acting benzodiazepine drug temazepam, as was based on the (self-reported) drug use information available in the corresponding clinical database. Such alignment seems explicable given that temazepam is often formulated as an oral capsule in which the active pharmaceutical ingredient is dispersed in short-chain polyethylene glycol (molecular weight below 1000 Da) to ensure a rapid release. This alignment also improved when using molecular evidence of (presumed) temazepam exposure, as relied on the identification of a phase II metabolite of this drug through spectral library matching (see Fig. S6 in the ESI†). A plausible explanation here is that temazepam is generally used on an ‘if needed’ (or: ‘*pro re nata’*, PRN) basis, while possibly also considering that it is a drug that is often misused and abused. It should be noted, however, that there is no perfect agreement between any of the two abovementioned temazepam exposure statuses and the intensity levels of signals in the peak clusters between 5 and 9 min (see Fig. S7 in the ESI†). Concretely, these peak clusters were not observed in some of the temazepam users, which should likely be viewed in the context that temazepam can also be supplied as an oral tablet which does not contain polyethylene glycol excipients. Still, we believe that the pharmaceutical excipient is a probable cause for the series of high-abundance peaks observed between 5 and 9 min in some of the urine samples.

Besides unusual peak clusters, some samples also featured a single, large, and broad peak at 12–12.5 min (see Fig. 1d) which caught our attention when visually inspecting the TICs. The corresponding mass spectra revealed a plethora of multiply-charged peaks which roughly corresponded to monoisotopic masses in the range of 3000 to 3500 Dalton (see Fig. S8 in the ESI†). The latter hinted towards usage of macrogol (*i.e.*, polyethylene glycol) 3350 oral laxative agents, which was supported by the (self-reported) drug use information available in the corresponding clinical database. However, agreement between the latter information and intensity levels of signals in the peak at 12–12.5 min is far from perfect (see Fig. S9 in the ESI†), as may be expected given that these laxative agents are often used on a PRN basis. Still, we believe that our findings may seem to disagree with the general understanding that macrogol laxative agents are not (or only in trace amounts) absorbed from the gastrointestinal tract.^16^

Altogether, the abovementioned findings suggest that pharmaceutical polymers and excipients could lead to (ten) thousands of high-abundance peaks being detected in clinical metabolomics datasets. As such, these peaks could potentially impact commonly used metabolomics data (pre)processing steps, in particular data normalization procedures, based on their high numbers and abundances but also by suppressing the signals of internal standards (see Fig. 1e). Thereby, pharmaceutical polymers and excipients may potentially affect the quantitative potential of LC-MS-based metabolomics, as will be discussed below.

### 3.2 Selection of exemplary quantifier signals

To assess the quantitative performance of the workflow that was used to obtain the data studied in this work, we selected two target analytes, namely the endogenous muscle breakdown product creatinine and the exogenous compound cotinine which is a phase I metabolite of nicotine. These compounds were the only two compounds which could both be detected in the metabolomics datasets and for which quantitative data was already available in the clinical database through prior performance of routine measurements in ISO 15189 certified laboratories.

**Fig. 2:**
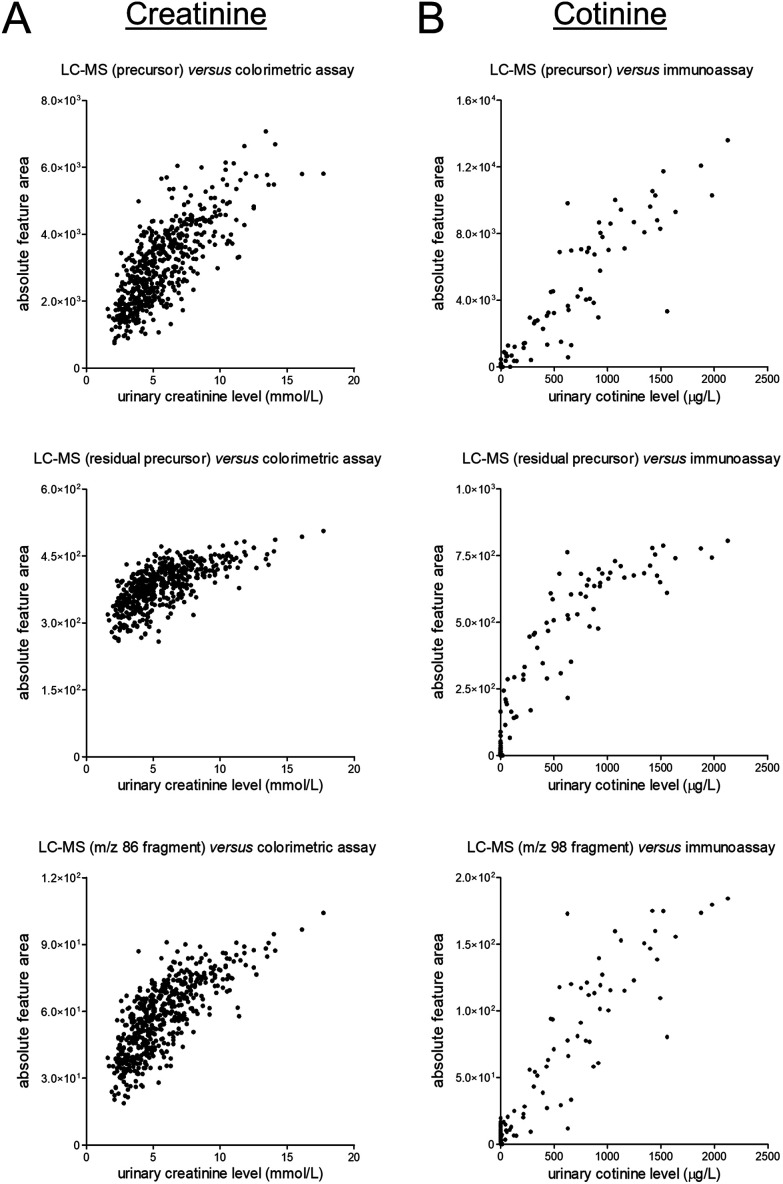
Scatter plots presenting unnormalized feature data of the MS1-level precursors (top), MS2-level residual precursors (middle), and representative MS2-level fragments (bottom) on the *y*-axis and the results of previously conducted routine measurements (in an ISO 15189 certified laboratory) on the *x*-axis for (A) the endogenous muscle breakdown product creatinine and (B) the exogenous phase I nicotine metabolite cotinine. The corresponding linear regression summary statistics for the MS1-level data are presented in Table S2.†

Next, we evaluated the correlations between (unnormalized) signals in the metabolomics dataset and the available ‘reference’ data (see Fig. 2). These evaluations yielded fairly linear correlations for the MS1-level precursor ions while various degrees of saturation were observed for the MS2-level residual precursor ions and representative MS2-level fragment ions. A possible explanation for this phenomenon is the use of the so-called ‘ion transmission control’ feature which could only be enabled for MS1-level acquisition on the MS instrument used to obtain the metabolomics data. Consequently, using the MS1-level precursor ions seems preferable for quantitative applications, although the linearity of certain lower-abundance MS2-level signals (*e.g.*, fragment ions, isotope peaks) demonstrated better linearity as compared to the MS2-level residual precursor ions (see Fig. S10 in the ESI†). Besides being useful for qualitative purposes, all MS2-level signals can furthermore be useful to assess specificity of the MS1-level precursor data, as can contribute to verifying data reliability.

**Fig. 3:**
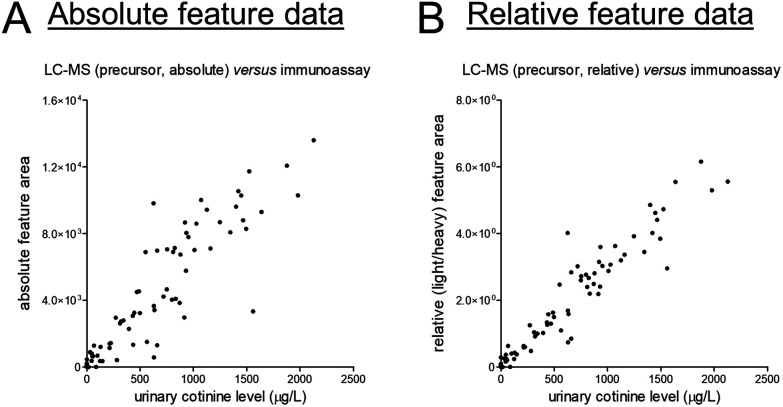
Scatter plots presenting unnormalized feature data of the (A) MS1-level precursor and (B) the light/heavy ratio of the MS1-level precursors on the *y*-axis and the results of previously conducted routine measurements (in an ISO 15189 certified laboratory) on the *x*-axis for the exogenous phase I nicotine metabolite cotinine. The corresponding linear regression summary statistics are presented in Table S2.†

At last, we selected creatinine and cotinine based on the presence of reference data, yet it should be acknowledged that reasons for disagreement between data from different assays come in plenty. For cotinine, we could yield some insights into such (potential) disagreement given that a deuterated version of cotinine was added to all samples as internal standard. This addition allowed us to correct for various sources of analytical variability, and the corresponding light-to-heavy (relative) feature ratios showed a linear and markedly stronger correlation with the reference data as compared to the absolute feature area (see Fig. 3). The latter data can, however, still be very useful for quantitative purposes, yet sufficient attention should be paid to statistical power in light of the magnitude of differences that can meaningfully be detected between study groups.^17^

### 3.3 Effect of normalization on exemplary quantifier signals

**Fig. 4:**
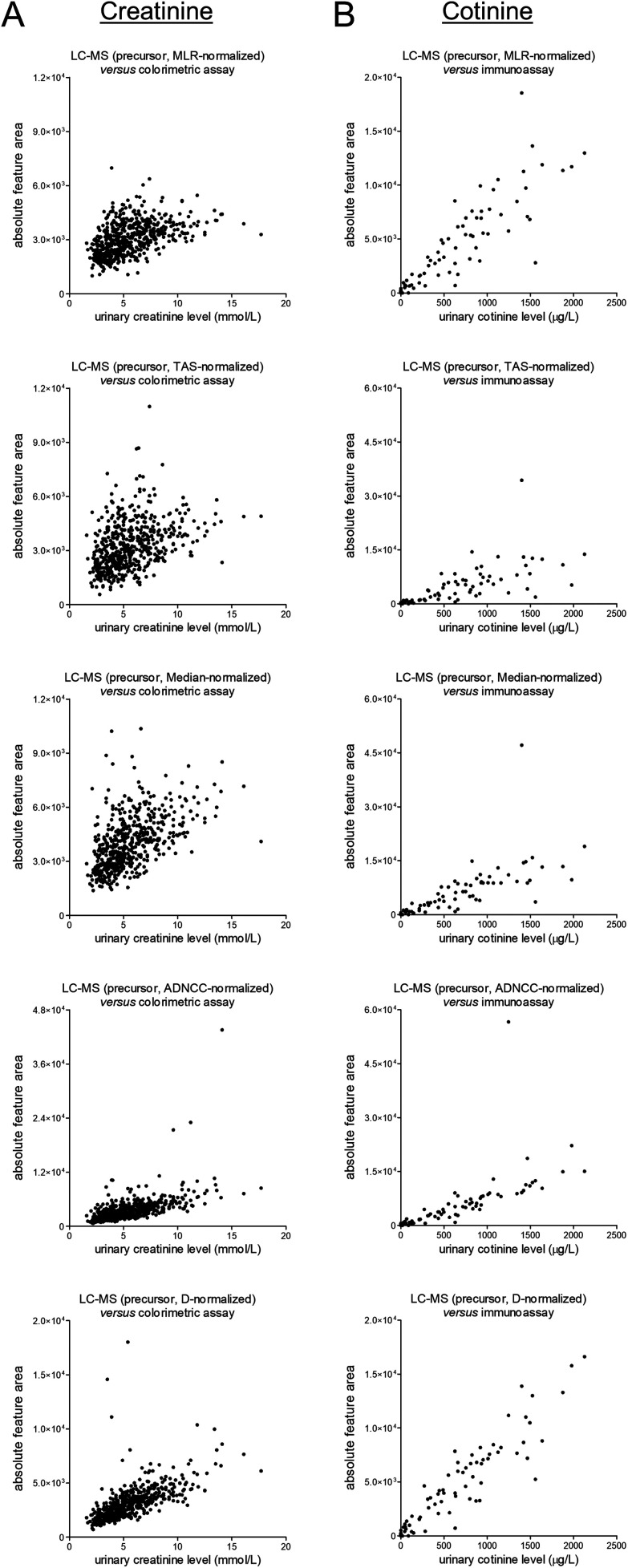
Scatter plots presenting differentially-normalized feature data of the MS1-level precursors (from top to bottom: MLR, TAS, Median, ADNCC, and D) on the *y*-axis and the results of previously-conducted routine measurements (in an ISO 15189 certified laboratory) on the *x*-axis for (A) the endogenous muscle breakdown product creatinine and (B) the exogenous phase I nicotine metabolite cotinine. The corresponding linear regression summary statistics are presented in Table S2.†

The unnormalized MS1-level data for creatinine and cotinine were scaled based on several normalization approaches, namely most likely ratio-based normalization (“MLR”),^14^ total area sums-based normalization (“TAS”), median peak ratios-based normalization (“Median”), normalization based on all five included internal standards (“ADNCC”), and normalization based on the diclofenac-^13^C_6_ internal standard only (“D”). The normalized data were subsequently plotted against the earlier-mentioned reference data, and the corresponding scatter plots are shown in Fig. 4. Upon comparison with matching plots for the unnormalized data, which were already shown in Fig. 2, all of the normalization procedures led to worsening of the correlations. All procedures furthermore led to (apparently) outlying results, although this effect seems less pronounced for MLR-based normalization.

Next, we aimed to assess whether the high-abundance peak clusters associated with the (presumed) exposure to CsA, temazepam, and macrogol 3350 contributed to the abovementioned worsening of the correlations, for example through the large numbers and high intensity levels of the respective peaks or by potentially suppressing the signals of internal standards. For this purpose, we provided views of the plots presented in Fig. 4 in which coloring is applied to indicate differential exposure statuses based on identified cyclosporine A, identified temazepam, and self-reported use of macrogol 3350 (see Fig. S11 and S22 in the ESI†). In our view, none of these plots seems to indicate a noticeable contribution of these exposures to the apparent worsening of the correlations when scaling the data, with the exception of ‘ADNCC’ multi-internal standard-based approach. In the corresponding figures (see Fig. S15 and S21 in the ESI†), one might notice some of the macrogol 3350-positive samples being outliers which warrants further investigation.

At last, in spite of not noticing a worrying effect of exposure to CsA, temazepam, and macrogol 3350 on data normalization, we would still feel inclined not to apply any normalization procedure to the studied clinical dataset, at least none of the studied approaches, given the findings depicted in Fig. 4. We are aware of other and more sophisticated normalization techniques, and we furthermore understand the necessity to normalize when pooling metabolomics data across studies.^18,19^ In addition, we acknowledge that typical metabolomics data processing pipelines include a data filtering step, often using the ‘80% rule’ which removes features that have missing data in more than 20% of the samples,^20^ before performing normalization. However, the use of normalization factors obtained after such a data filtering procedure did not seem to affect the observed correlations that much, at least not for this dataset (see Fig. S23 in the ESI†).

## 4 Conclusions

This work provides a possible explanation for the unexpected observation put forward by two previously published studies that usage of cyclosporine A, an immunosuppressive drug which should predominantly be excreted in the bile, was responsible for the most pronounced clustering in principal component analysis of urinary metabolomics data from kidney transplant recipients. Specifically, we found series of high-abundance peaks with 14 and 44 Dalton differences which likely originate from the lipid-based pharmaceutical excipient used to enhance oral delivery of this drug. Comparable series were also found in the urine of temazepam users, and the corresponding signals likely originate from the polymeric pharmaceutical ingredient polyethylene glycol that is used to ensure a rapid release of this short-acting benzodiazepine drug. In addition, we found clusters of thousands of high-abundance peaks corresponding to monoisotopic masses in the range of 3000 to 3500 Dalton. These findings hinted towards usage of macrogol 3350 oral laxative agents, as was supported by available (self-reported) drug use information. However, it should be noted that such an explanation goes against the consensus that these agents are not (or only in trace amounts) absorbed from the gastrointestinal tract and thus are not expected in urine.

Besides describing the potential presence of pharmaceutical polymers and excipients in clinical metabolomics data, our work also aimed to assess the potential implications of their presence on the quantitative performance of LC-MS-based small-molecule profiling workflows. In particular, we put emphasis on the selected data normalization techniques which did not lead to more reliable quantitative data, at least not in our opinion and not for the dataset we studied. Data normalization is, however, an important step in metabolomics data processing pipelines, and we believe that our findings can help to optimize existing and/or develop novel data normalization approaches to be applied to clinical metabolomics datasets.

## Author contributions

The manuscript was written through contributions of all authors. All authors have given approval to the final version of the manuscript. Conceptualization – F. K, G. H.; methodology – F. K.; formal analysis – F. K.; investigation – F. K., F. B. N., P. K-R.; resources – S. J. L. B., T. I., G. H.; data curation – F. K.; writing – original draft – F. K.; writing – review & editing – F. K., F. B. N., S. J. L. B., P. K-R., G. H.; visualization – F. K.; supervision – F. K., S. J. L. B., T. I., P. K-R., G. H.; funding acquisition – F. K., S. J. L. B., T. I., G. H.

## Conflicts of interest

There are no conflicts to declare.

## Supplementary Material

AN-149-D3AN01874A-s001
