# Supplementary material for: High-abundance peaks and peak clusters associate with pharmaceutical polymers and excipients in urinary untargeted clinical metabolomics data: exploration of their origin and possible impact on label-free quantification
Source: Analyst. 2024 Jan 3;149(4):1061–7. doi: 10.1039/d3an01874a (PMC10866140; doi:10.1039/d3an01874a)
Supplement: AN-149-D3AN01874A-s001 [file AN-149-D3AN01874A-s001.pdf]

Electronic Supplementary Material (ESI) for Analyst.
This journal is © The Royal Society of Chemistry 2024

Supplementary Information

High-abundance peaks and peak clusters associate with pharmaceutical polymers and excipients in urinary untargeted clinical metabolomics data: *exploration of their origin and possible impact on label-free quantification*

Frank Klont,* Fleur B. Nijdam, Stephan J.L. Bakker, Pekka Keski-Rahkonen, Gérard Hopfgartner, and TransplantLines Investigators

**E-mail address: frank.klont@rug.nl (F. Klont).*

Table S1. Overview of MarkerView data (pre)processing settings.

Setting	Value
Feature finding:	
Experiment	MS1-only or all MS2 experiments
Minimum retention time	0.50 min
Maximum retention time	16.00 min
Subtraction offset	15 scans
Subtraction multiplication factor	1.3
Noise threshold	5
Minimum spectral peak width	5 ppm
Minimum retention time peak width	5 scans
Assign charge states	Enabled
Feature alignment:	
Retention time tolerance	0.50 min
Mass tolerance	0.01 Da
Feature filtering:	
Maximum number of peaks	8,000,000*
Remove peaks in < N samples	Disabled
Isotope filtering	Disabled
Intensity threshold	5
Use exclusion list	Disabled
Retention time filtering	Disabled
Use area integrated from raw data, not from original peak finding	Disabled
*Data normalization**:*	
Normalization method 0	Disabled
Normalization method 1	Most-Likely Ratio (“MLR”)
Normalization method 2	Total Area Sums (“TAS”)
Normalization method 3	Median Peak Ratios (“Median”)
Normalization method 4	Internal Standards (“ADNCC”)
	Acetaminophen/paracetamol-D_4 (m/z 156.096, 5.60 min)
	Diclofenac-$^{13}C_6$ (m/z 302.044, 13.40 min)
	Naproxen-D_3 (m/z 234.120, 12.30 min)
	Caffeine-$^{13}C_3$ (m/z 198.098, 7.30 min)
	Cotinine-D_3 (m/z 180.121, 4.50 min)
Normalization method 5	Internal Standards (“D”)
	Diclofenac-$^{13}C_6$ (m/z 302.044, 13.40 min)
Principle component analysis:	
PCA preprocessing - Weighting	None
PCA preprocessing - Scaling	Pareto
Perform PCA-DA (supervised)	Disabled
Principle component analysis-discriminant analysis:	
PCA preprocessing - Weighting	None
PCA preprocessing - Scaling	Pareto
Perform PCA-DA (supervised)	Enabled
T-test:	
Samples per group for “first to last” comparison	Disabled
Use Welch t-test	Disabled

*: This parameter was set high enough to prevent peaks from getting filtered at this stage.
**: If performed, normalization was performed after feature peak list generation.

Table S2. Overview of linear regression summary statistics relating to the data presented in the Figures 2, 3, and 4, which compare differentially normalized MS1 feature level data of creatinine and cotinine with quantitative data of both compounds as were obtained through routine measurements conducted in an ISO 15189 certified laboratory.

		Fit		Slope	
**Normalization approach**	**N**	**R^2**	**Sy.x**	**F**	**P-value**
creatinine:					
unnormalized	550	0.59	753	780	<0.0001
MLR	550	0.21	802	148	<0.0001
MLR after 80% filtering	550	0.24	859	175	<0.0001
TAS	550	0.15	1227	96	<0.0001
TAS after 80% filtering	550	0.10	1106	64	<0.0001
Median	550	0.29	1215	223	<0.0001
Median after 80% filtering	550	0.25	1390	188	<0.0001
ADNCC	550	0.34	2218	279	<0.0001
D	550	0.40	1236	365	<0.0001
cotinine:					
unnormalized	384	0.90	749	3562	<0.0001
cotinine-D_3 internal standard	384	0.96	0.22	8490	<0.0001
MLR	384	0.87	908	2576	<0.0001
MLR after 80% filtering	384	0.87	979	2614	<0.0001
TAS	384	0.66	1730	757	<0.0001
TAS after 80% filtering	384	0.66	1658	758	<0.0001
Median	384	0.70	2097	885	<0.0001
Median after 80% filtering	384	0.58	3086	521	<0.0001
ADNCC	384	0.63	2479	644	<0.0001
D	384	0.93	697	4871	<0.0001

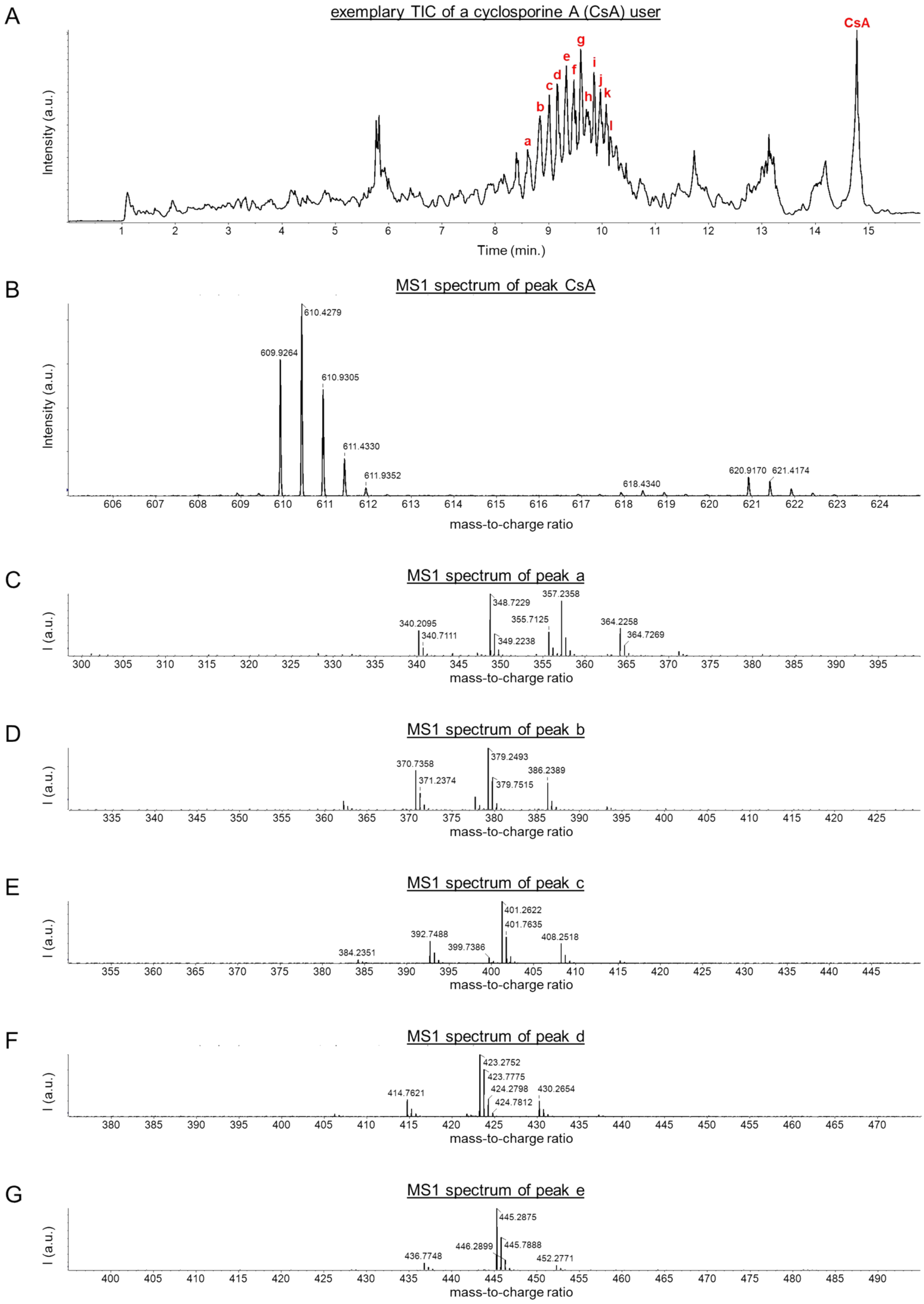

Figure S1. (**A**) Total ion current chromatogram (TIC) and (**B**-**N**) selected MS1 spectra presumably reflecting polyethoxylated castor oil of a user of the immunosuppressive drug cyclosporine A.

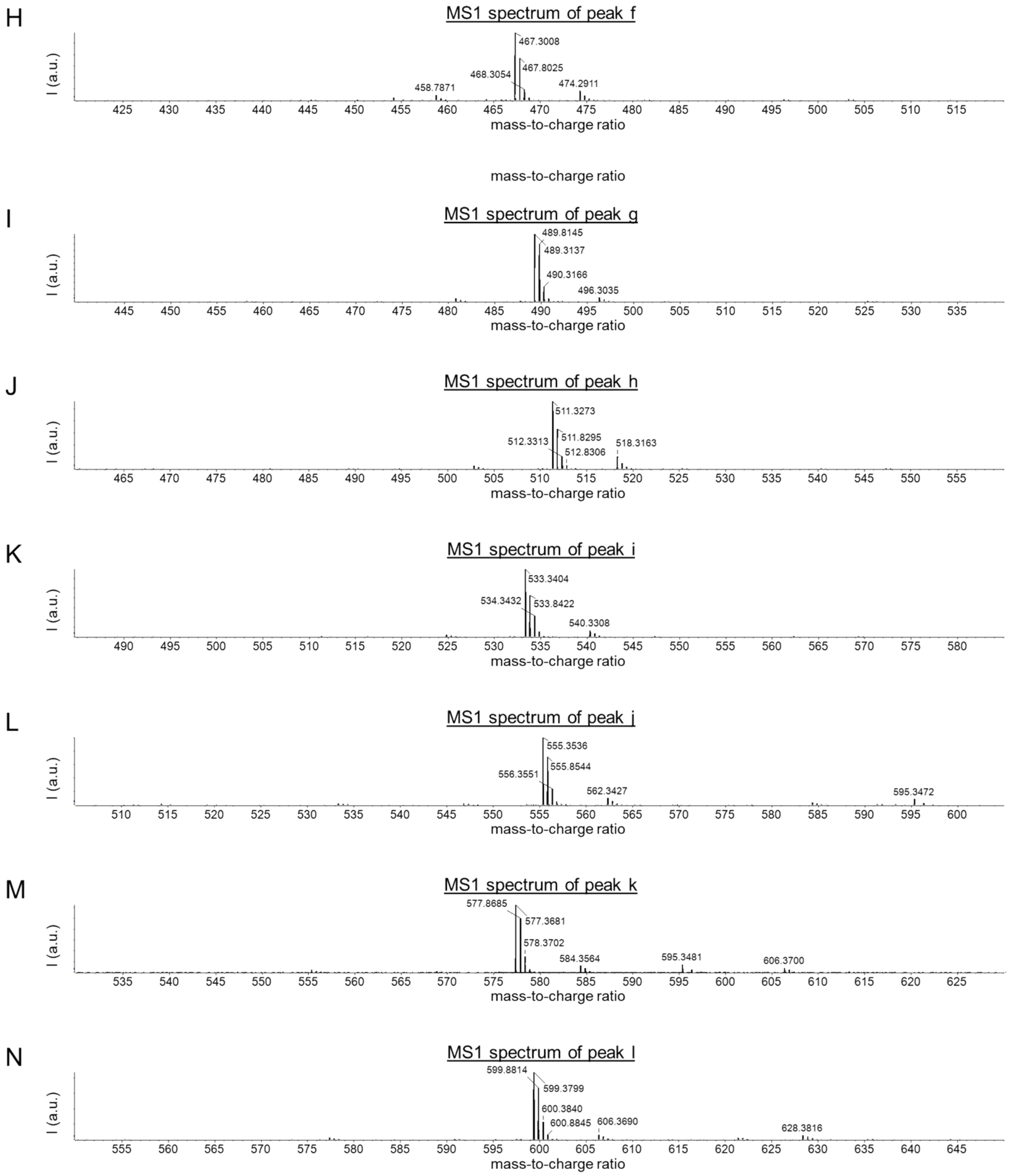

Figure S1. Cont.

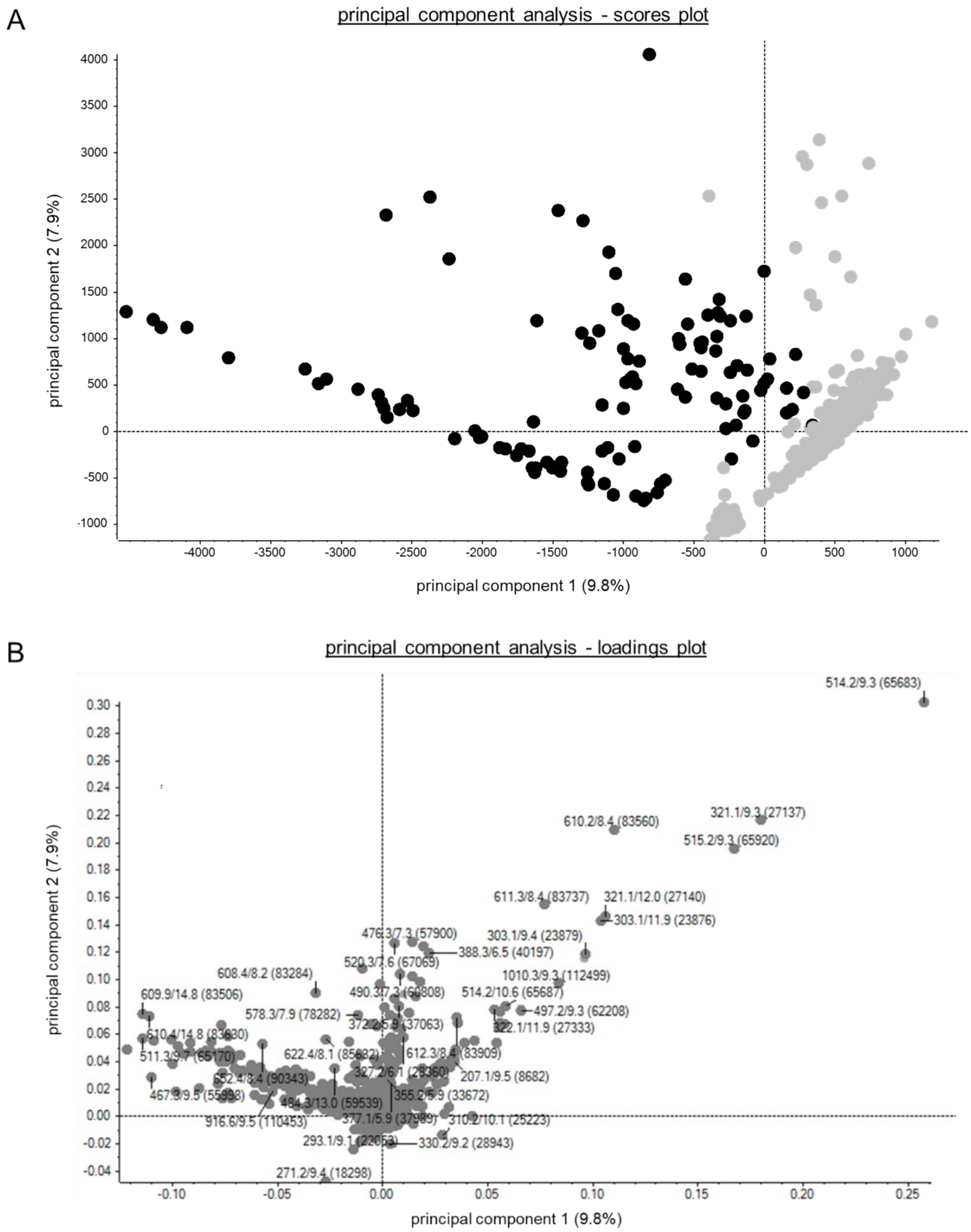

Figure S2. Pareto-scaled (**A**) scores and (**B**) loadings plots for unsupervised principal component analysis of unnormalized MS1-level feature data of 570 stable kidney transplant recipients. In pane A, users of cyclosporine A are indicated in black and nonusers are indicated in light grey, as reflect analytically-confirmed exposure statuses. Regarding the latter, this analytical conformation concerns a level 1 metabolite identification according to the Metabolomics Standards Initiative (L.W. Sumner, *et al*. Metabolomics, 2007, 3, 211-221) for which we used a cyclosporine A reference standard (Sigma Aldrich, Cat. No. PHR1092; CID 49867938) and a commercial software tool (*i.e.,* SCIEX PeakView, version 2.2.0.11391) using previously-published settings (F. Klont, *et al*. J Clin Epidemiol, 2021, 135, 10-16).

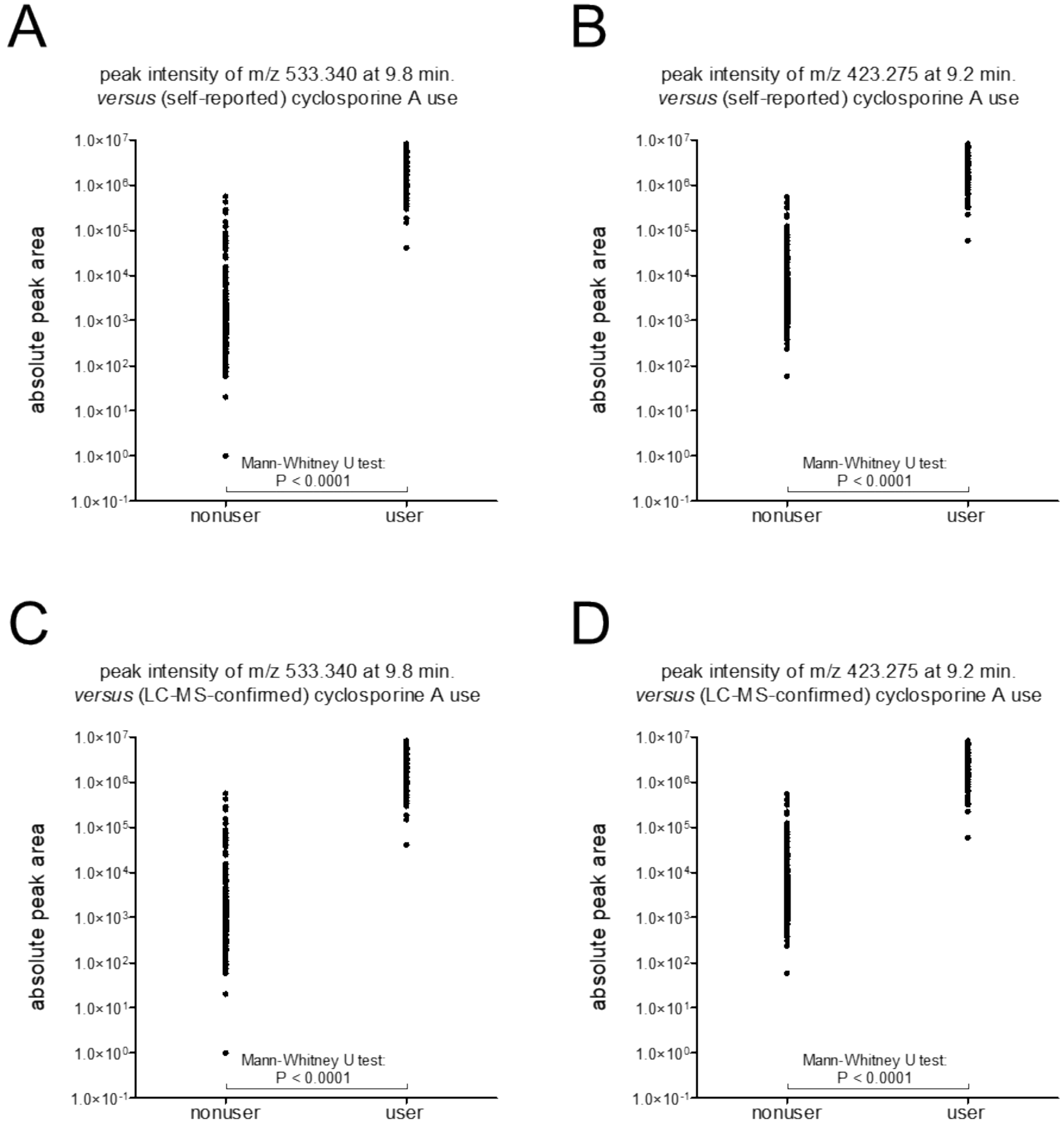

Figure S3. Scatter plots presenting unnormalized LC-MS peak areas of two MS1-level signals associated with (presumed) exposure to cyclosporine A capsules, as is presented on the y-axis, and usage of cyclosporine A, as is presented on the x-axis. With respect to the two signals, (**A**, **C**) the first one reflects the most intense peak presented in Fig. S1K (peak 'i' in Fig. S1A), and (**B**,**D**) the second one reflects the most intense peak presented in Fig. S1F (peak 'd' in Fig. S1A). With respect to the exposure status, this reflects (**A**,**B**) self-reported drug use or (**C**,**D**) analytically-confirmed presence of the drug of interest in the corresponding urine sample. In case of cyclosporine A, such analytical conformation concerns a level 1 metabolite identification according to the Metabolomics Standards Initiative (L.W. Sumner, *et al*. Metabolomics, 2007, 3, 211-221) for which we used a cyclosporine A reference standard (Sigma Aldrich, Cat. No. PHR1092; CID 49867938) and a commercial software tool (*i.e.,* SCIEX PeakView, version 2.2.0.11391) using previously-published settings (F. Klont, *et al*. J Clin Epidemiol, 2021, 135, 10-16).

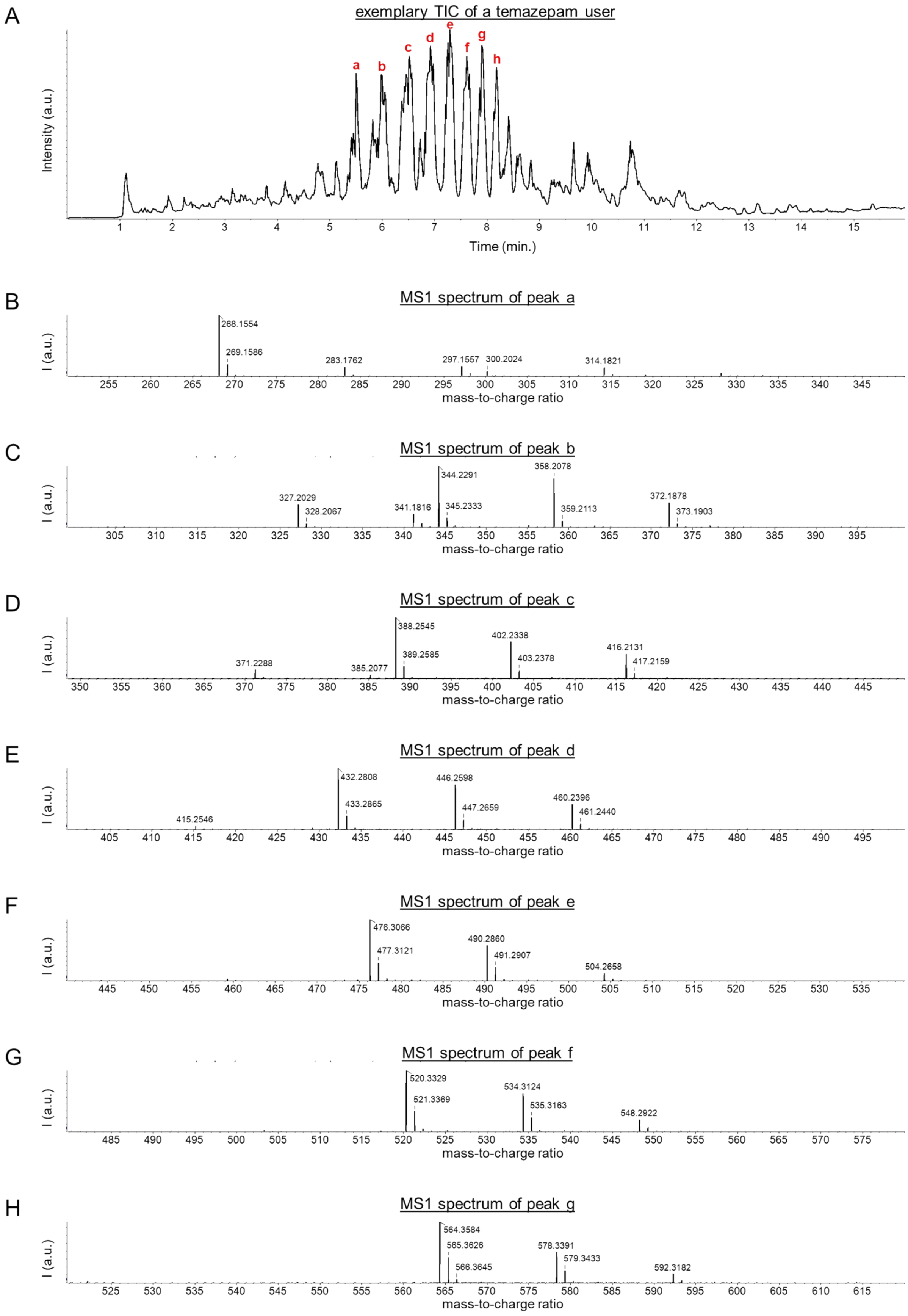

Figure S4. (**A**) Total ion current chromatogram (TIC) and (**B**-**I**) selected MS1 spectra reflecting polyethylene glycol of a user of the short-acting benzodiazepine drug temazepam.

I

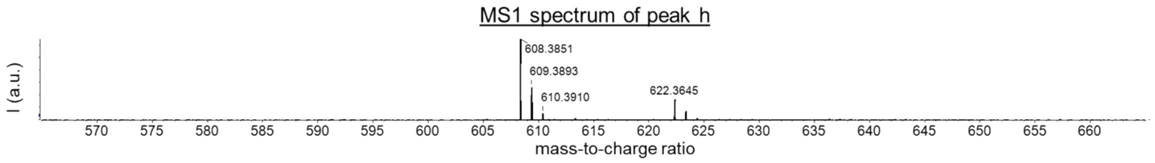

Figure S4. Cont.

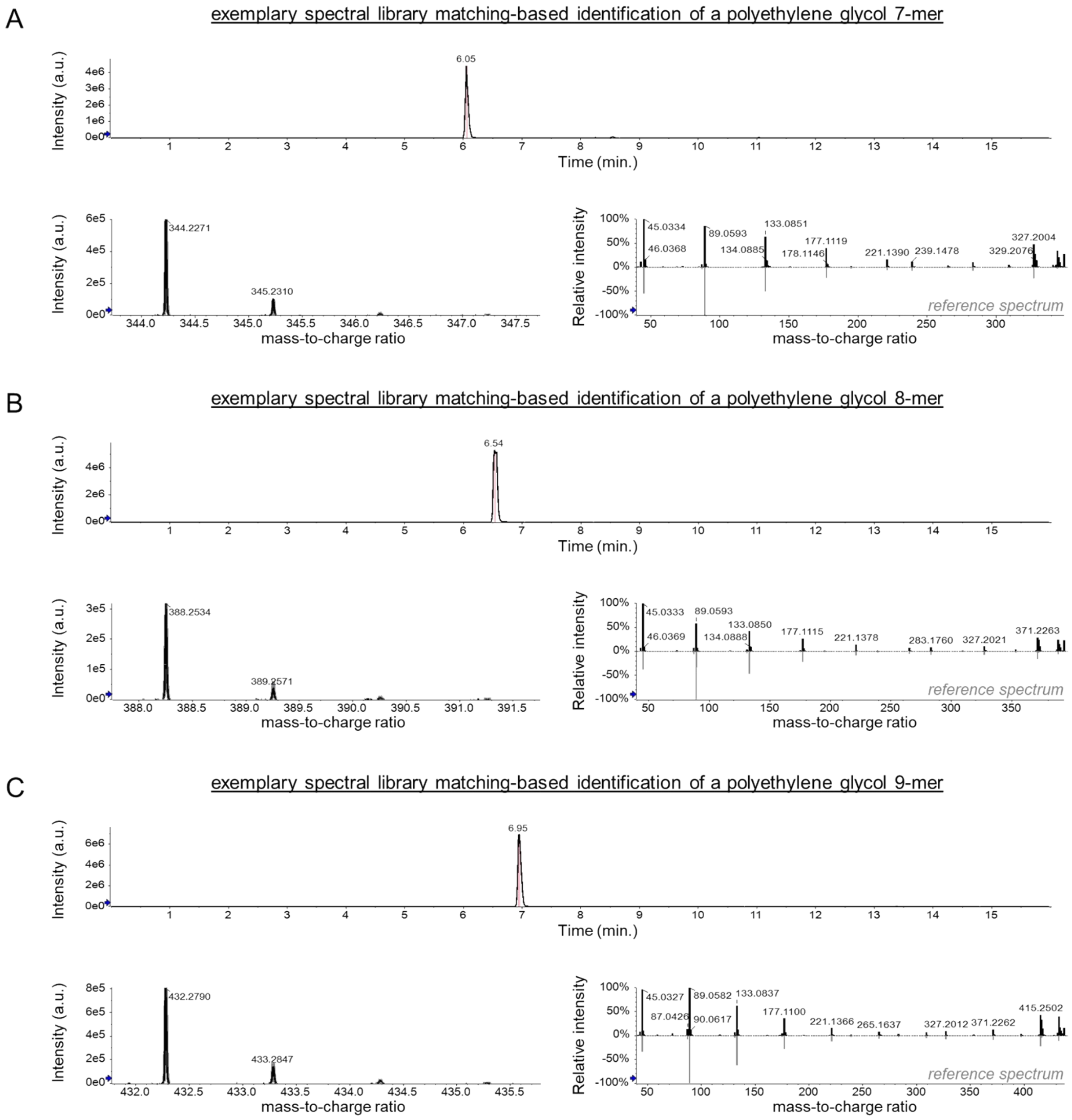

Figure S5. Exemplary spectral library matching-based identification of ammonium adducts of short-chain (7- to 12-mer) polyethylene glycol molecules (CID not available because polyethylene glycol has no discrete structure), as were observed in the urine of a transplant recipient who declared usage of the short-acting benzodiazepine drug temazepam. Regarding the data obtained and presented, these concern level 2 metabolite identifications according to the Metabolomics Standards Initiative (L.W. Sumner, *et al*. Metabolomics, 2007, 3, 211-221) for which we employed a commercial spectral library (*i.e.,* SCIEX 'Forensic', version 1.1) and a commercial software tool (*i.e.,* SCIEX PeakView, version 2.2.0.11391) using previously-published settings (F. Klont, *et al*. J Clin Epidemiol, 2021, 135, 10-16).

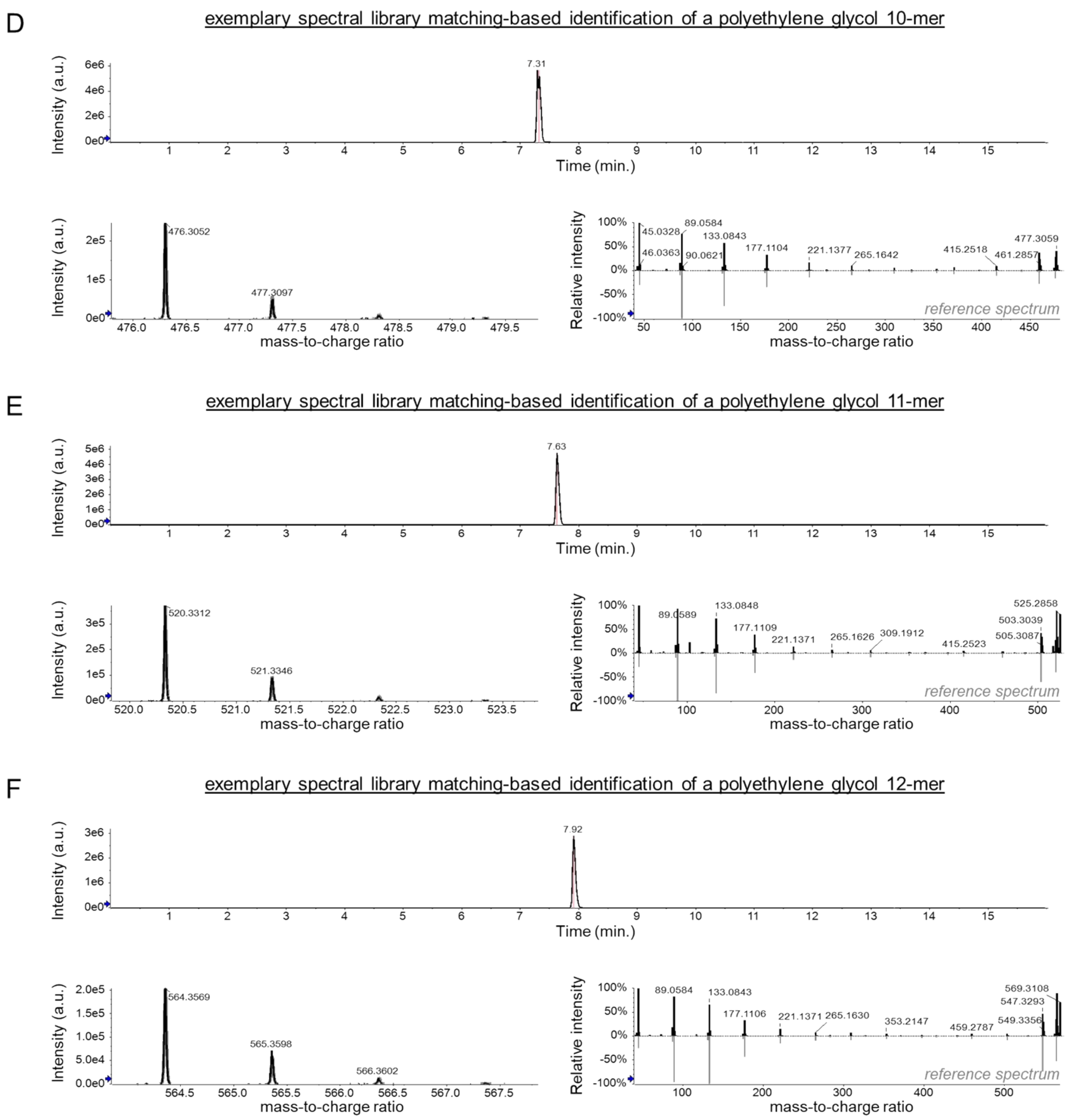

Figure S5. Cont.

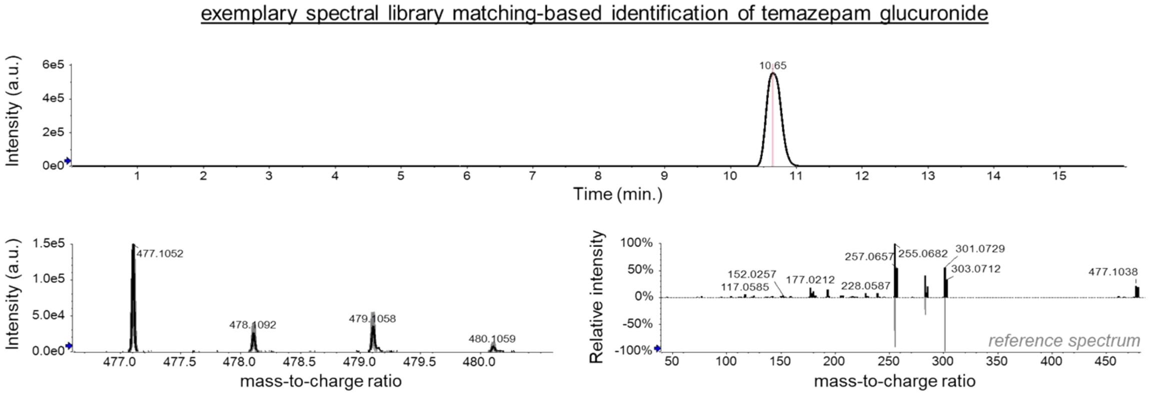

Figure S6. Exemplary spectral library matching-based identification of temazepam glucuronide (CID 76973794), which is a phase II metabolite of the short-acting benzodiazepine drug temazepam and which was observed in the urine of a transplant recipient who declared usage of this drug. Regarding the data obtained and presented, these concern level 2 metabolite identifications according to the Metabolomics Standards Initiative (L.W. Sumner, *et al*. Metabolomics, 2007, 3, 211-221) for which we employed a commercial spectral library (*i.e.,* SCIEX 'Forensic', version 1.1) and a commercial software tool (*i.e.,* SCIEX PeakView, version 2.2.0.11391) using previously-published settings (F. Klont, *et al*. J Clin Epidemiol, 2021, 135, 10-16).

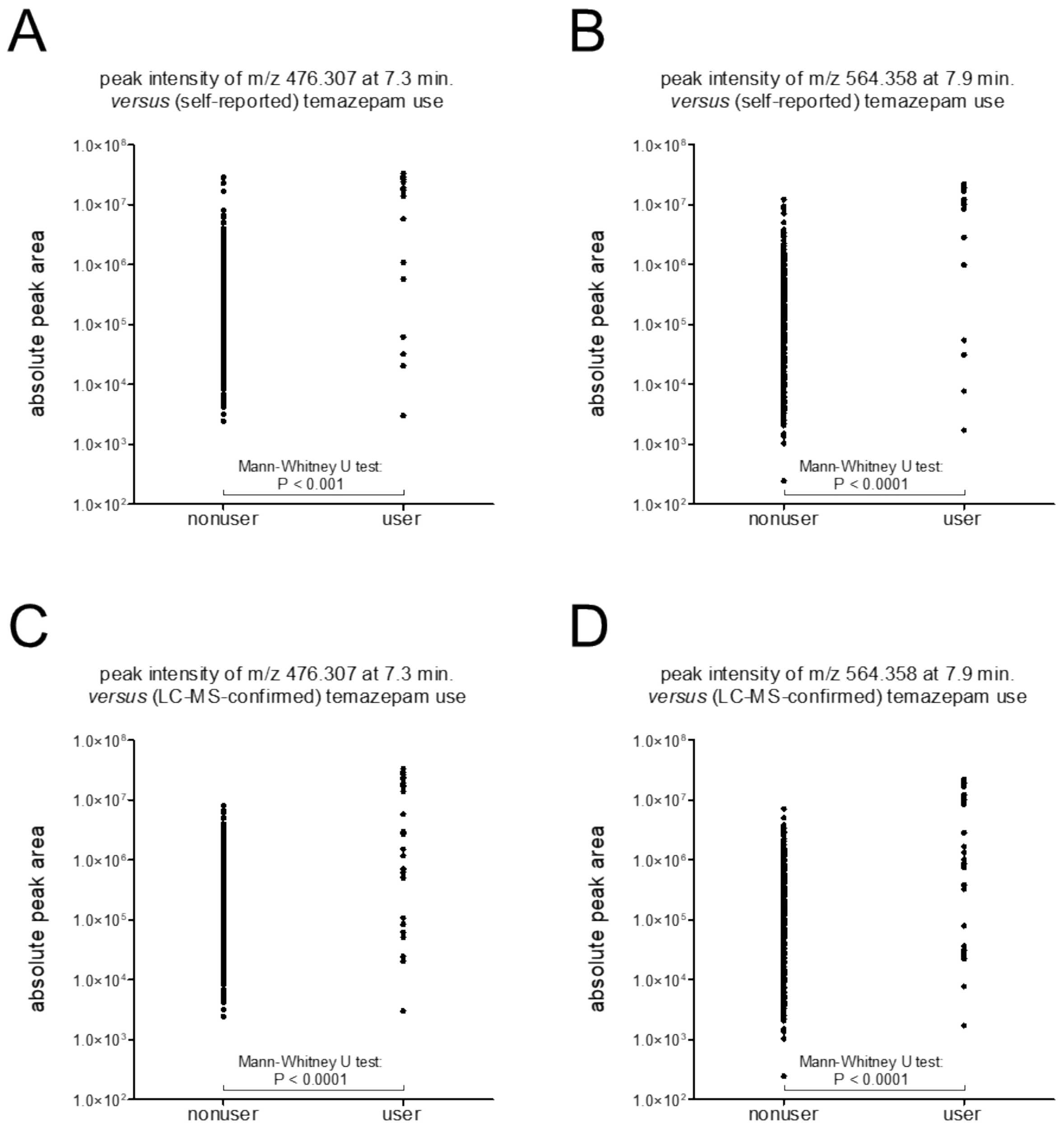

Figure S7. Scatter plots presenting unnormalized LC-MS peak areas of two MS1-level signals associated with (presumed) exposure to temazepam, as is presented on the y-axis, and usage of temazepam, as is presented on the x-axis. With respect to the two signals, (**A**, **C**) the first one reflects the most intense peak presented in Fig. S4F (peak 'e' in Fig. S4A), and (**B**,**D**) the second one reflects the most intense peak presented in Fig. S4H (peak 'g' in Fig. S4A). With respect to the exposure status, this reflects (**A**,**B**) self-reported drug use or (**C**,**D**) analytically-confirmed presence of the drug of interest in the corresponding urine sample. In case of temazepam, such analytical conformation concerns the identification of temazepam glucuronide (CID 76973794) which reflects a level 2 metabolite identification according to the Metabolomics Standards Initiative (L.W. Sumner, *et al*. Metabolomics, 2007, 3, 211-221) for which we employed a commercial spectral library (*i.e.,* SCIEX 'Forensic', version 1.1) and a commercial software tool (*i.e.,* SCIEX PeakView, version 2.2.0.11391) using previously-published settings (F. Klont, *et al*. J Clin Epidemiol, 2021, 135, 10-16).

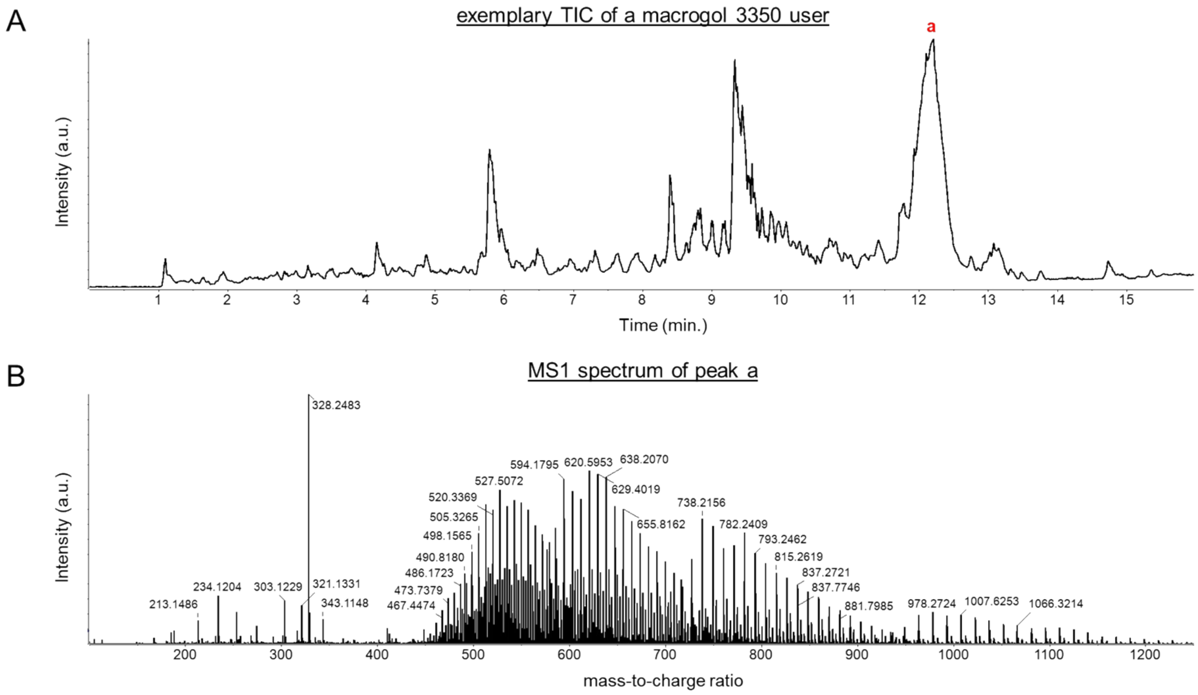

Figure S8. (**A**) Total ion current chromatogram (TIC) and (**B**) a selected MS1 spectrum of a self-declared user of the laxative agent macrogol 3350.

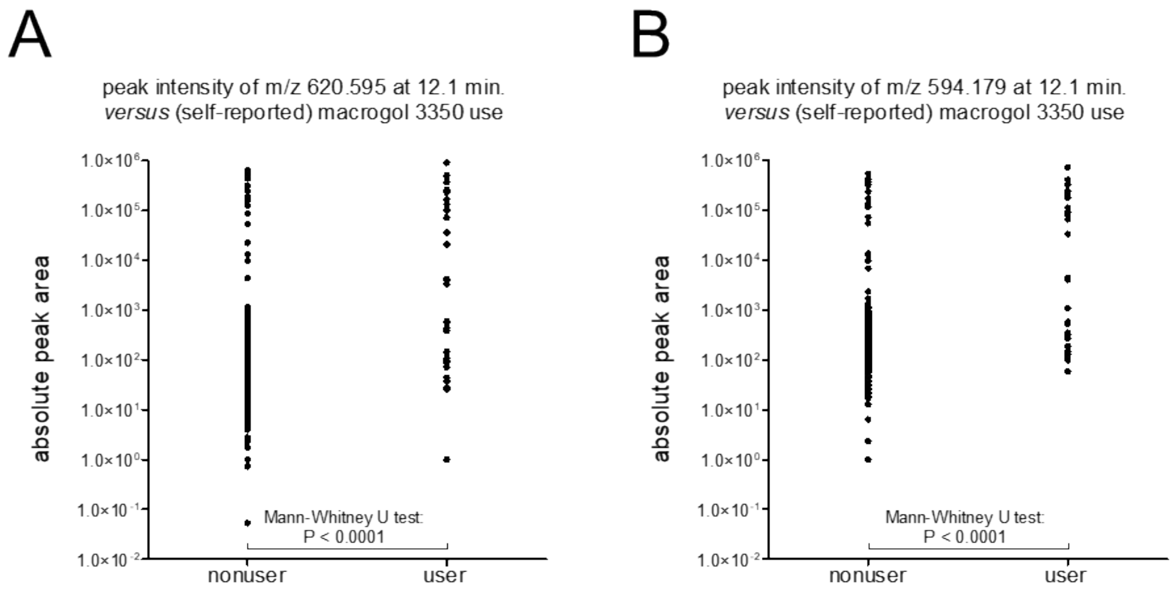

Figure S9. Scatter plots presenting unnormalized LC-MS peak areas of two MS1-level signals associated with (presumed) exposure to macrogol 3350, as is presented on the y-axis, and self-reported usage of macrogol 3350, as is presented on the x-axis. With respect to the two signals, both are among the most intense peaks presented in Fig. S8B.

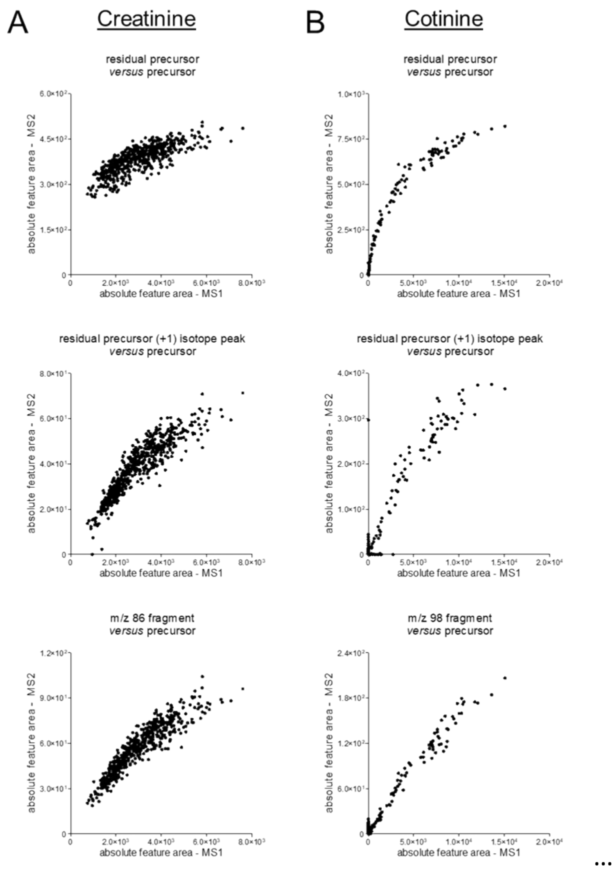

Figure S10. Scatter plots presenting unnormalized feature data of the MS2-level residual precursors (top), the first isotope peaks of the MS2-level residual precursors (middle), and representative MS2-level fragments (bottom) on the y-axis and unnormalized feature data of the MS1-level precursors on the x-axis for (**A**) the endogenous muscle breakdown product creatinine and (**B**) the exogenous phase I nicotine metabolite cotinine.

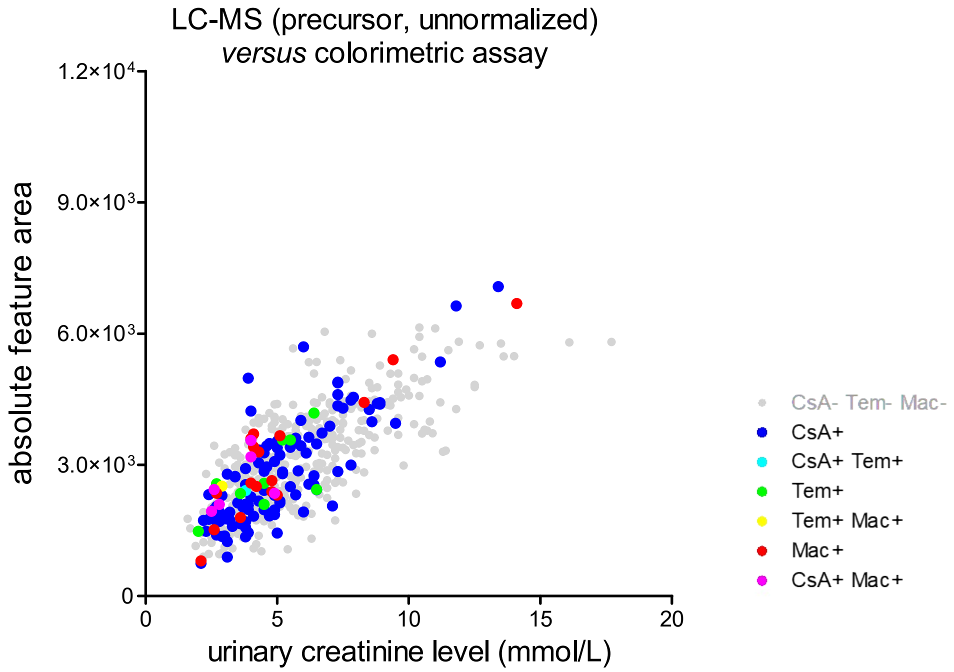

Figure S11. Scatter plot presenting unnormalized feature data of the MS1-level precursor on the y-axis and the results of previously-conducted routine measurements (in an ISO 15189 certified laboratory) on the x-axis for the endogenous muscle breakdown product creatinine. In this figure, coloring is applied to indicate differential exposure statuses based on identified cyclosporine A (‘CsA’; see Fig. S3C), identified temazepam (‘Tem’; see Fig. S7C), and self-reported use of macrogol 3350 (‘Mac’; see Fig. 9A). With respect to the latter, no analytical confirmation was possible for this pharmaceutical polymer, and the data presented in Fig. S9 show several nonusers with considerably high (presumed) polymer signals. Hence, we classified nonusers with peak areas for both signals presented in Fig. S9 above an arbitrary cutoff of 20,000 a.u. as ‘Mac+’ as well in the presented figure.

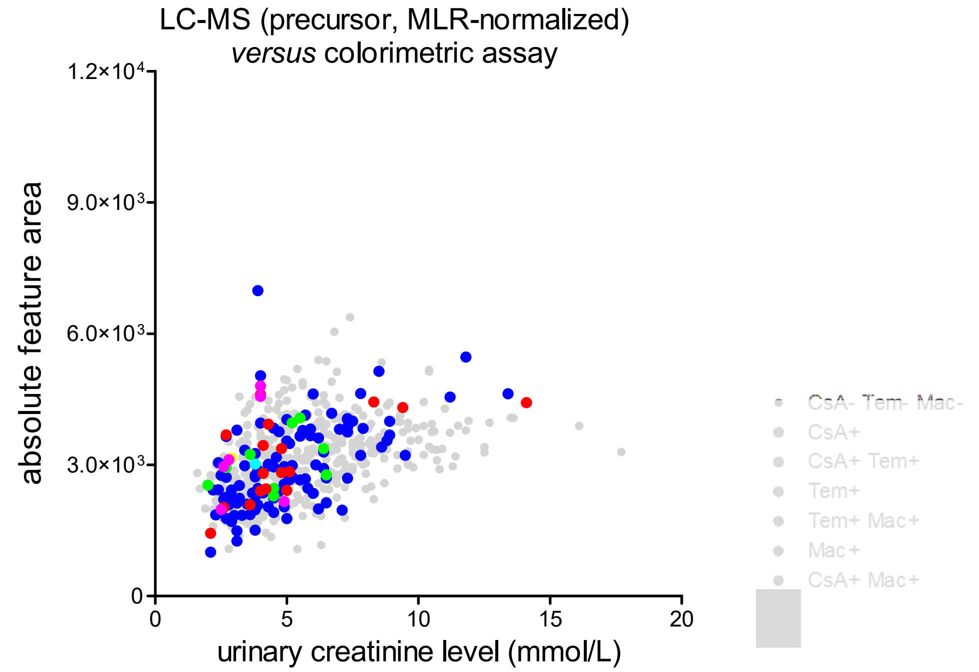

Figure S12. Scatter plot presenting MLR-normalized feature data of the MS1-level precursor on the y-axis and the results of previously-conducted routine measurements (in an ISO 15189 certified laboratory) on the x-axis for the endogenous muscle breakdown product creatinine. In this figure, coloring is applied to indicate differential exposure statuses based on identified cyclosporine A ('CsA'; see Fig. S3C), identified temazepam ('Tem'; see Fig. S7C), and self-reported use of macrogol 3350 ('Mac'; see Fig. 9A). With respect to the latter, no analytical confirmation was possible for this pharmaceutical polymer, and the data presented in Fig. S9 show several nonusers with considerably high (presumed) polymer signals. Hence, we classified nonusers with peak areas for both signals presented in Fig. S9 above an arbitrary cutoff of 20,000 a.u. as 'Mac+' as well in the presented figure.

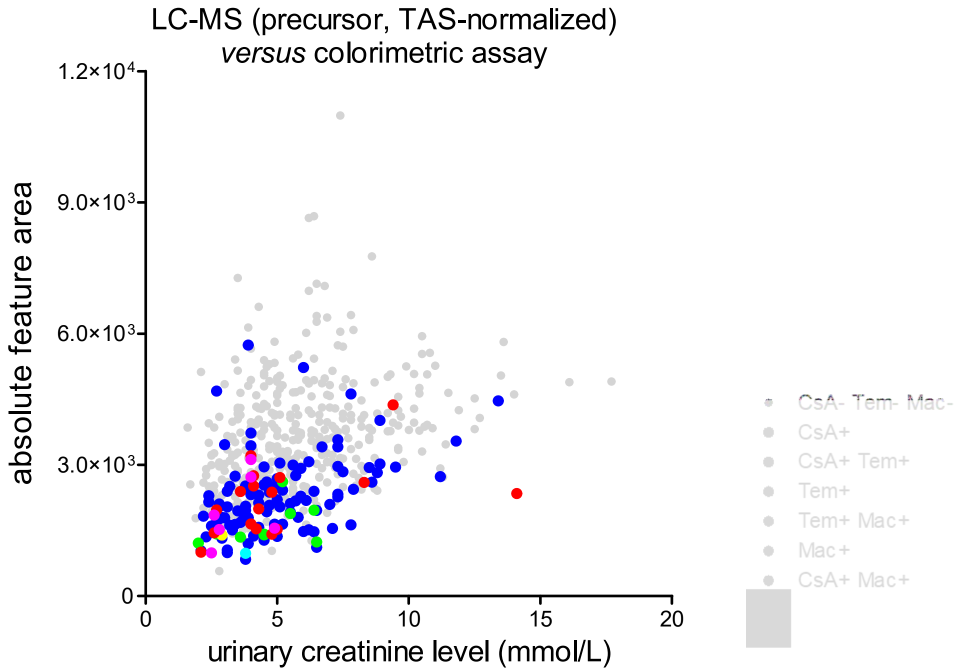

Figure S13. Scatter plot presenting TAS-normalized feature data of the MS1-level precursor on the y-axis and the results of previously-conducted routine measurements (in an ISO 15189 certified laboratory) on the x-axis for the endogenous muscle breakdown product creatinine. In this figure, coloring is applied to indicate differential exposure statuses based on identified cyclosporine A ('CsA'; see Fig. S3C), identified temazepam ('Tem'; see Fig. S7C), and self-reported use of macrogol 3350 ('Mac'; see Fig. 9A). With respect to the latter, no analytical confirmation was possible for this pharmaceutical polymer, and the data presented in Fig. S9 show several nonusers with considerably high (presumed) polymer signals. Hence, we classified nonusers with peak areas for both signals presented in Fig. S9 above an arbitrary cutoff of 20,000 a.u. as 'Mac+' as well in the presented figure.

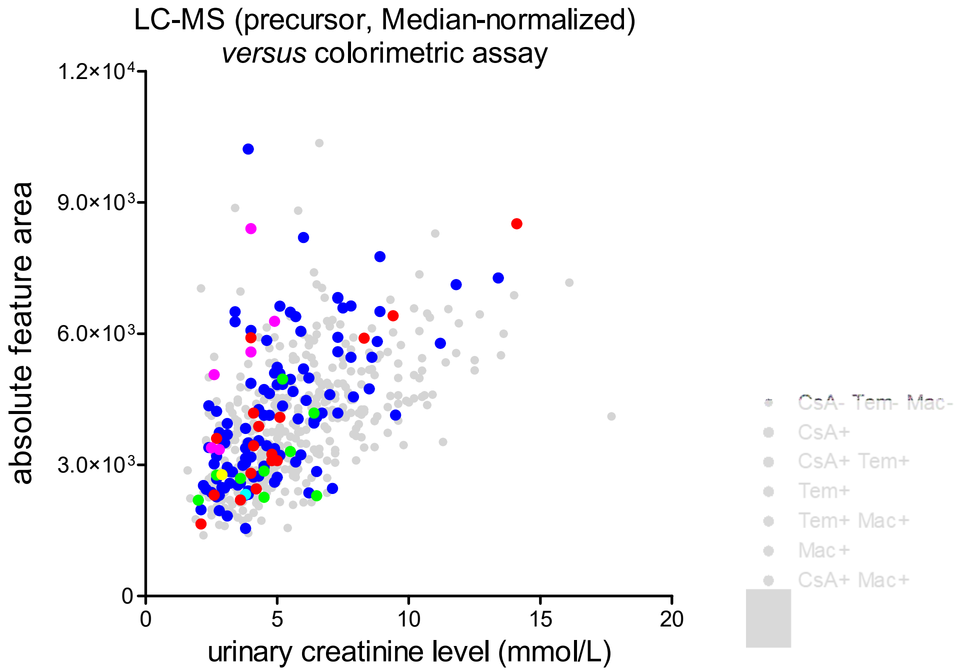

Figure S14. Scatter plot presenting Median-normalized feature data of the MS1-level precursor on the y-axis and the results of previously-conducted routine measurements (in an ISO 15189 certified laboratory) on the x-axis for the endogenous muscle breakdown product creatinine. In this figure, coloring is applied to indicate differential exposure statuses based on identified cyclosporine A ('CsA'; see Fig. S3C), identified temazepam ('Tem'; see Fig. S7C), and self-reported use of macrogol 3350 ('Mac'; see Fig. 9A). With respect to the latter, no analytical confirmation was possible for this pharmaceutical polymer, and the data presented in Fig. S9 show several nonusers with considerably high (presumed) polymer signals. Hence, we classified nonusers with peak areas for both signals presented in Fig. S9 above an arbitrary cutoff of 20,000 a.u. as 'Mac+' as well in the presented figure.

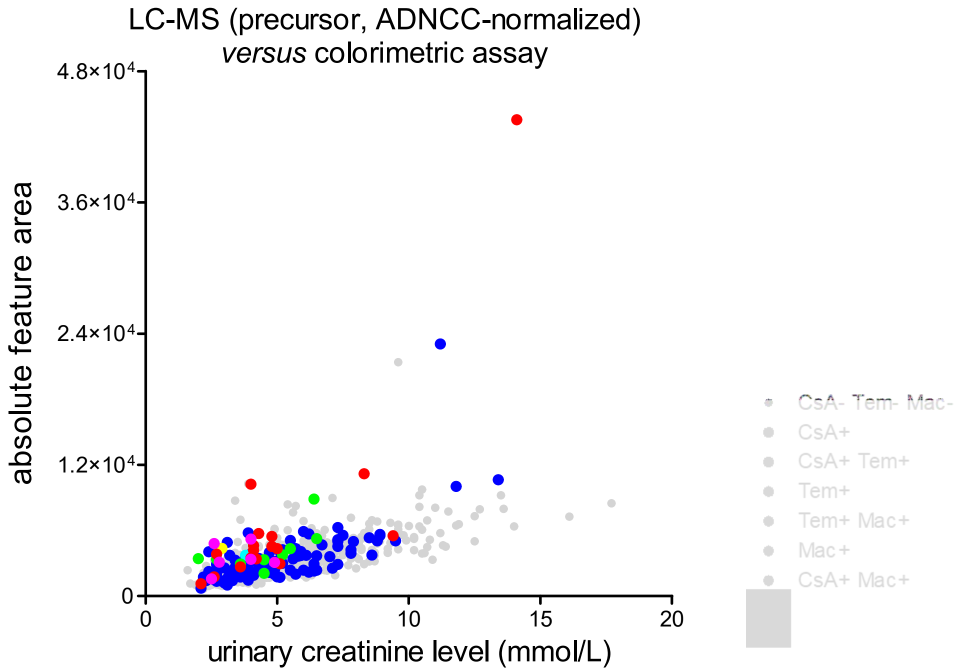

Figure S15. Scatter plot presenting ADNCC-normalized feature data of the MS1-level precursor on the y-axis and the results of previously-conducted routine measurements (in an ISO 15189 certified laboratory) on the x-axis for the endogenous muscle breakdown product creatinine. In this figure, coloring is applied to indicate differential exposure statuses based on identified cyclosporine A ('CsA'; see Fig. S3C), identified temazepam ('Tem'; see Fig. S7C), and self-reported use of macrogol 3350 ('Mac'; see Fig. 9A). With respect to the latter, no analytical confirmation was possible for this pharmaceutical polymer, and the data presented in Fig. S9 show several nonusers with considerably high (presumed) polymer signals. Hence, we classified nonusers with peak areas for both signals presented in Fig. S9 above an arbitrary cutoff of 20,000 a.u. as 'Mac+' as well in the presented figure.

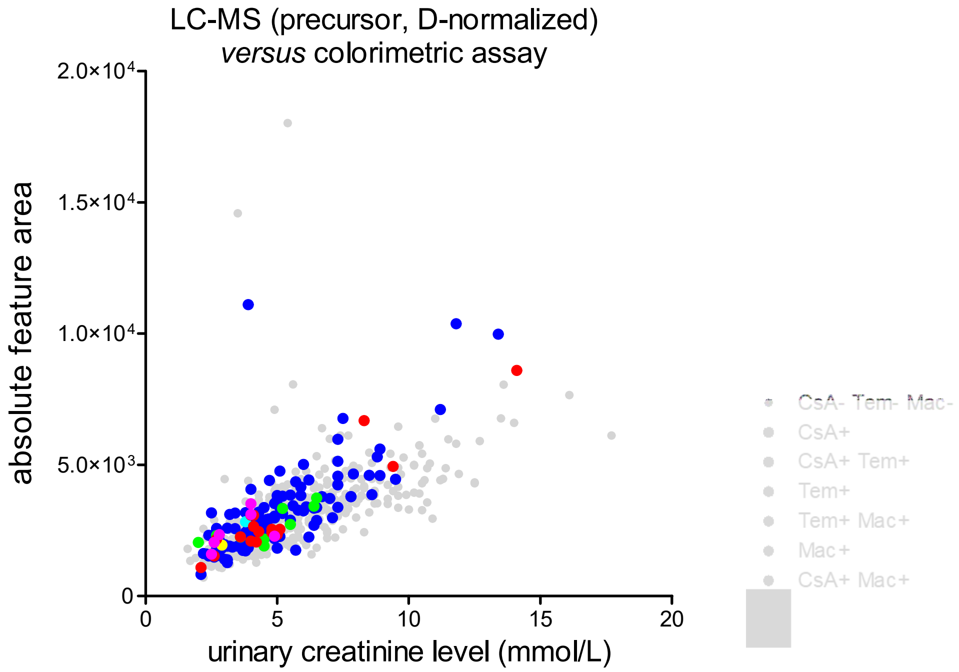

Figure S16. Scatter plot presenting D-normalized feature data of the MS1-level precursor on the y-axis and the results of previously-conducted routine measurements (in an ISO 15189 certified laboratory) on the x-axis for the endogenous muscle breakdown product creatinine. In this figure, coloring is applied to indicate differential exposure statuses based on identified cyclosporine A ('CsA'; see Fig. S3C), identified temazepam ('Tem'; see Fig. S7C), and self-reported use of macrogol 3350 ('Mac'; see Fig. 9A). With respect to the latter, no analytical confirmation was possible for this pharmaceutical polymer, and the data presented in Fig. S9 show several nonusers with considerably high (presumed) polymer signals. Hence, we classified nonusers with peak areas for both signals presented in Fig. S9 above an arbitrary cutoff of 20,000 a.u. as 'Mac+' as well in the presented figure.

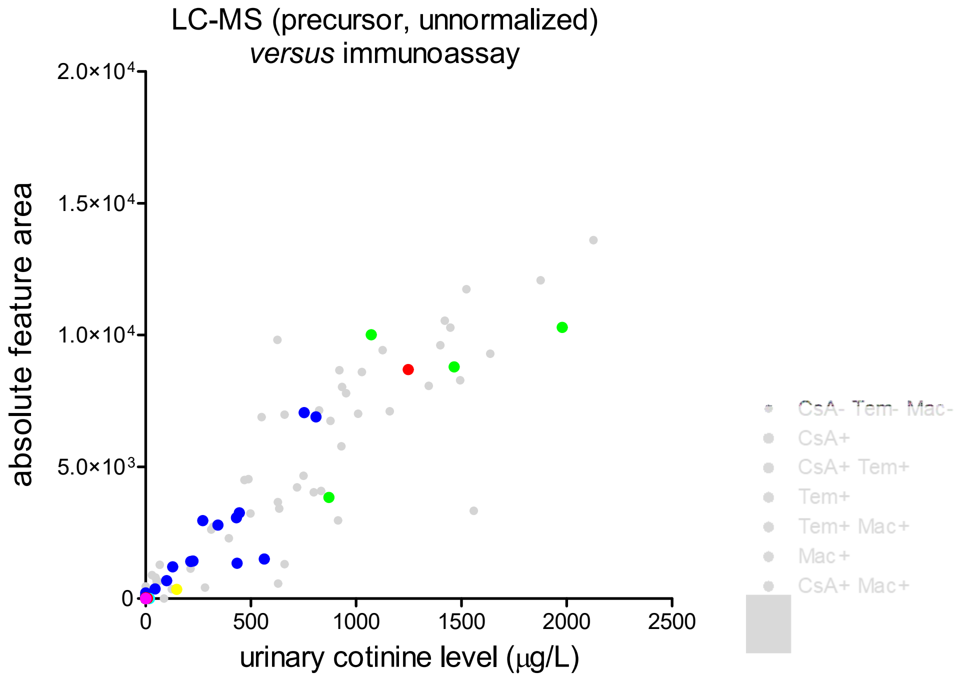

Figure S17. Scatter plot presenting unnormalized feature data of the MS1-level precursor on the y-axis and the results of previously-conducted routine measurements (in an ISO 15189 certified laboratory) on the x-axis for the exogenous phase I nicotine metabolite cotinine. In this figure, coloring is applied to indicate differential exposure statuses based on identified cyclosporine A ('CsA'; see Fig. S3C), identified temazepam ('Tem'; see Fig. S7C), and self-reported use of macrogol 3350 ('Mac'; see Fig. 9A). With respect to the latter, no analytical confirmation was possible for this pharmaceutical polymer, and the data presented in Fig. S9 show several nonusers with considerably high (presumed) polymer signals. Hence, we classified nonusers with peak areas for both signals presented in Fig. S9 above an arbitrary cutoff of 20,000 a.u. as 'Mac+' as well in the presented figure.

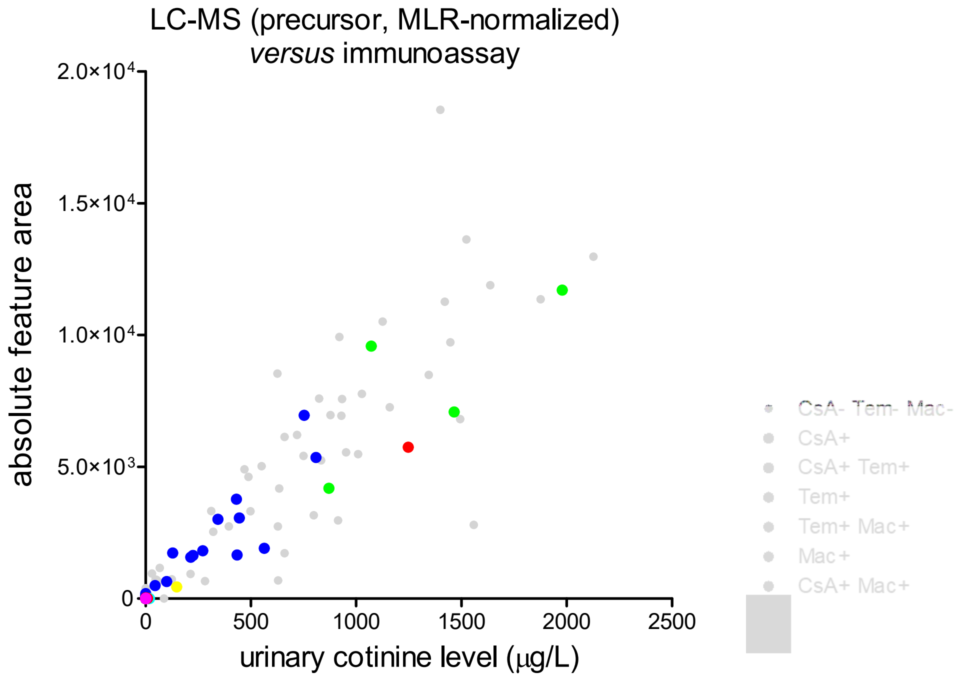

Figure S18. Scatter plot presenting MLR-normalized feature data of the MS1-level precursor on the y-axis and the results of previously-conducted routine measurements (in an ISO 15189 certified laboratory) on the x-axis for the exogenous phase I nicotine metabolite cotinine. In this figure, coloring is applied to indicate differential exposure statuses based on identified cyclosporine A ('CsA'; see Fig. S3C), identified temazepam ('Tem'; see Fig. S7C), and self-reported use of macrogol 3350 ('Mac'; see Fig. 9A). With respect to the latter, no analytical confirmation was possible for this pharmaceutical polymer, and the data presented in Fig. S9 show several nonusers with considerably high (presumed) polymer signals. Hence, we classified nonusers with peak areas for both signals presented in Fig. S9 above an arbitrary cutoff of 20,000 a.u. as 'Mac+' as well in the presented figure.

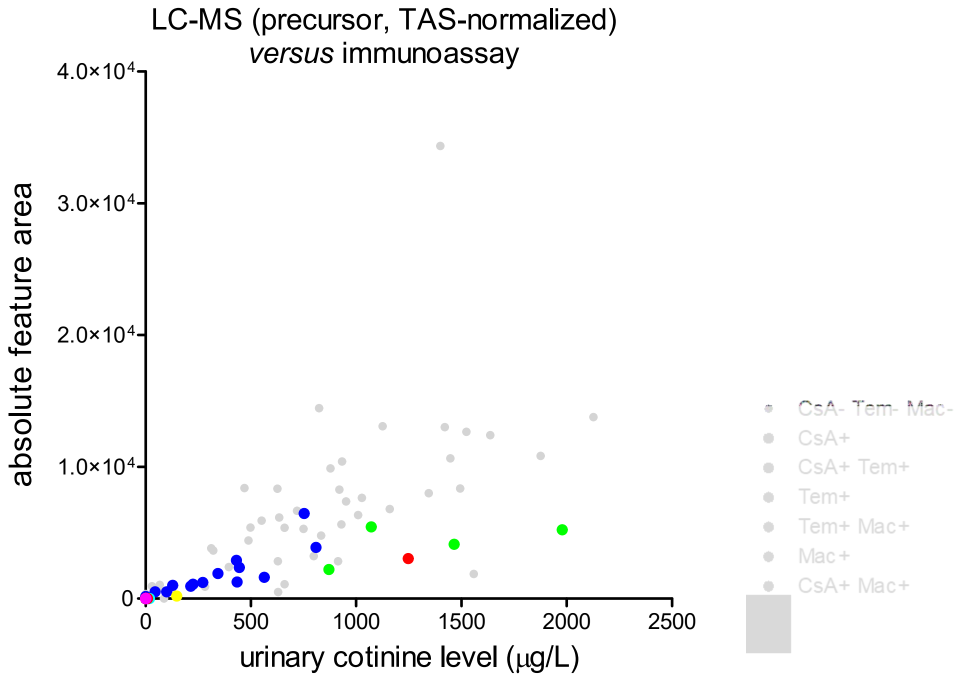

Figure S19. Scatter plot presenting TAS-normalized feature data of the MS1-level precursor on the y-axis and the results of previously-conducted routine measurements (in an ISO 15189 certified laboratory) on the x-axis for the exogenous phase I nicotine metabolite cotinine. In this figure, coloring is applied to indicate differential exposure statuses based on identified cyclosporine A ('CsA'; see Fig. S3C), identified temazepam ('Tem'; see Fig. S7C), and self-reported use of macrogol 3350 ('Mac'; see Fig. 9A). With respect to the latter, no analytical confirmation was possible for this pharmaceutical polymer, and the data presented in Fig. S9 show several nonusers with considerably high (presumed) polymer signals. Hence, we classified nonusers with peak areas for both signals presented in Fig. S9 above an arbitrary cutoff of 20,000 a.u. as 'Mac+' as well in the presented figure.

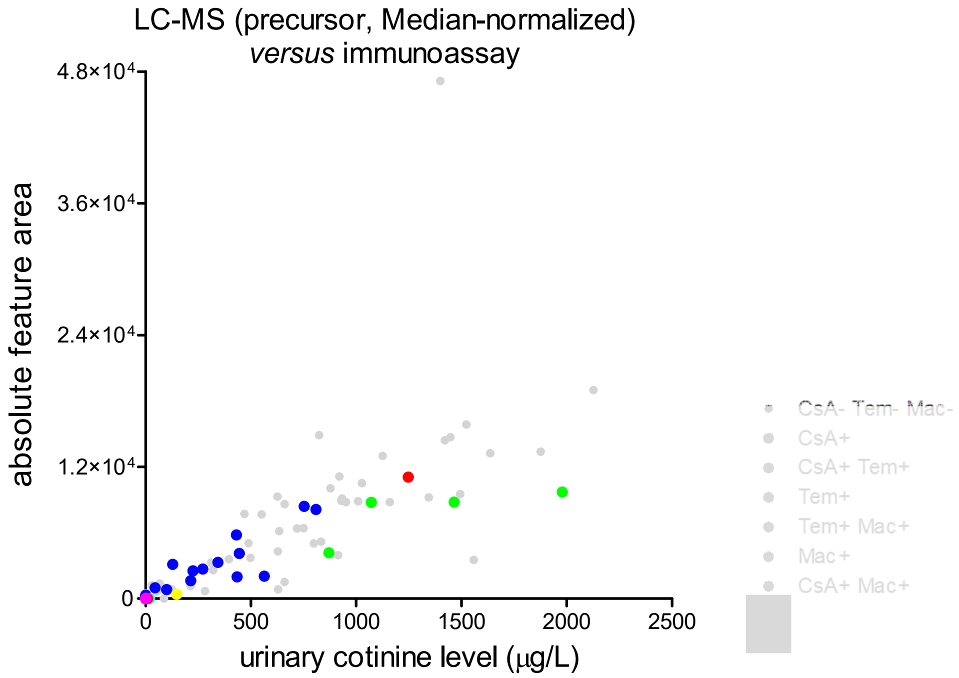

Figure S20. Scatter plot presenting Median-normalized feature data of the MS1-level precursor on the y-axis and the results of previously-conducted routine measurements (in an ISO 15189 certified laboratory) on the x-axis for the exogenous phase I nicotine metabolite cotinine. In this figure, coloring is applied to indicate differential exposure statuses based on identified cyclosporine A ('CsA'; see Fig. S3C), identified temazepam ('Tem'; see Fig. S7C), and self-reported use of macrogol 3350 ('Mac'; see Fig. 9A). With respect to the latter, no analytical confirmation was possible for this pharmaceutical polymer, and the data presented in Fig. S9 show several nonusers with considerably high (presumed) polymer signals. Hence, we classified nonusers with peak areas for both signals presented in Fig. S9 above an arbitrary cutoff of 20,000 a.u. as 'Mac+' as well in the presented figure.

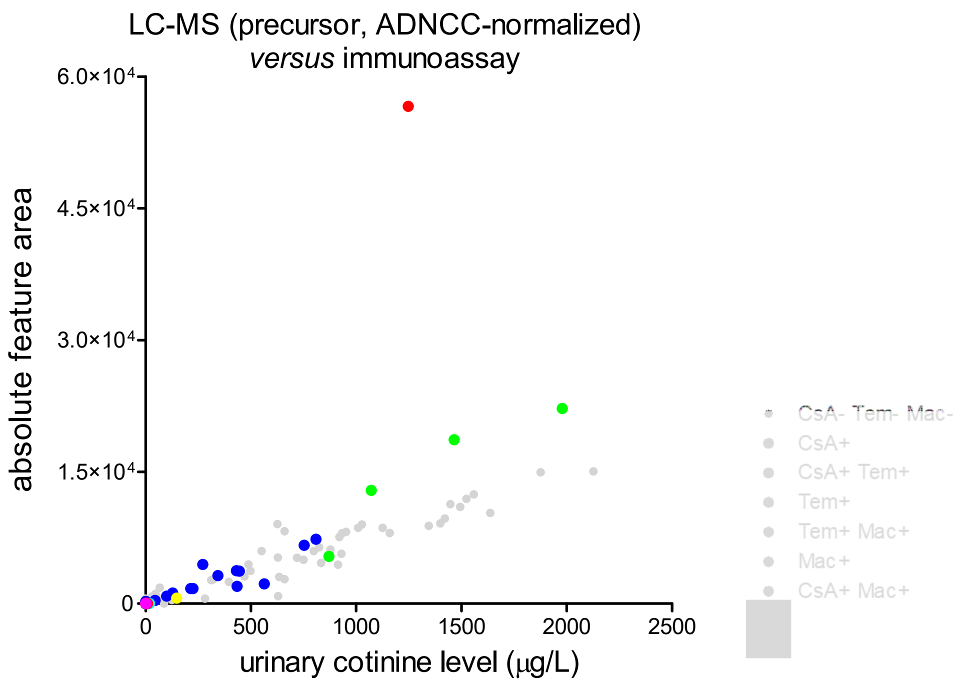

Figure S21. Scatter plot presenting ADNCC-normalized feature data of the MS1-level precursor on the y-axis and the results of previously-conducted routine measurements (in an ISO 15189 certified laboratory) on the x-axis for the exogenous phase I nicotine metabolite cotinine. In this figure, coloring is applied to indicate differential exposure statuses based on identified cyclosporine A (‘CsA’; see Fig. S3C), identified temazepam (‘Tem’; see Fig. S7C), and self-reported use of macrogol 3350 (‘Mac’; see Fig. 9A). With respect to the latter, no analytical confirmation was possible for this pharmaceutical polymer, and the data presented in Fig. S9 show several nonusers with considerably high (presumed) polymer signals. Hence, we classified nonusers with peak areas for both signals presented in Fig. S9 above an arbitrary cutoff of 20,000 a.u. as ‘Mac+’ as well in the presented figure.

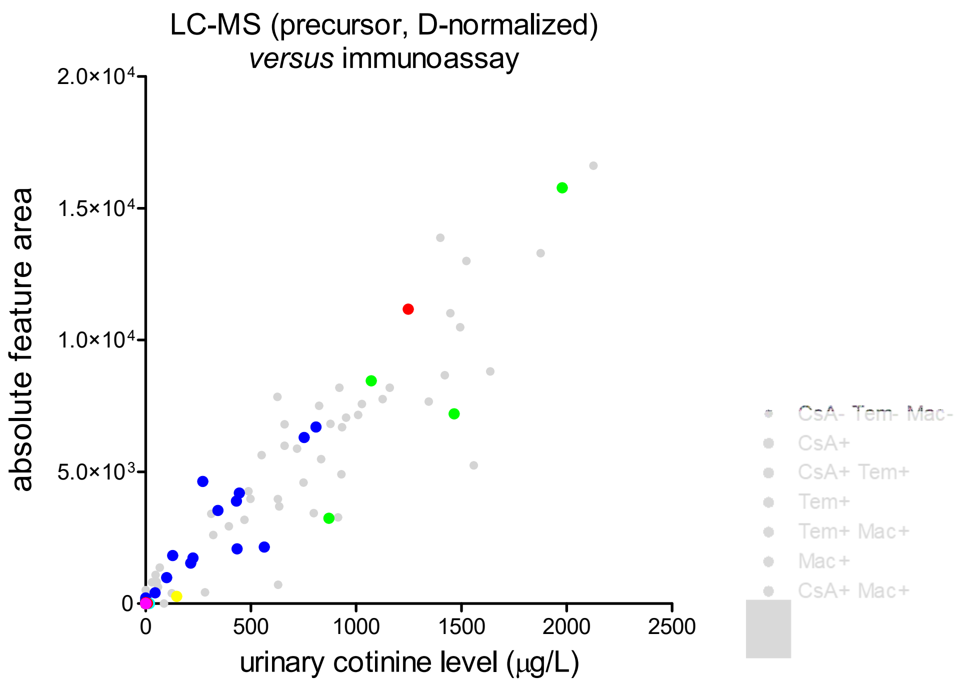

Figure S22. Scatter plot presenting D-normalized feature data of the MS1-level precursor on the y-axis and the results of previously-conducted routine measurements (in an ISO 15189 certified laboratory) on the x-axis for the exogenous phase I nicotine metabolite cotinine. In this figure, coloring is applied to indicate differential exposure statuses based on identified cyclosporine A ('CsA'; see Fig. S3C), identified temazepam ('Tem'; see Fig. S7C), and self-reported use of macrogol 3350 ('Mac'; see Fig. 9A). With respect to the latter, no analytical confirmation was possible for this pharmaceutical polymer, and the data presented in Fig. S9 show several nonusers with considerably high (presumed) polymer signals. Hence, we classified nonusers with peak areas for both signals presented in Fig. S9 above an arbitrary cutoff of 20,000 a.u. as 'Mac+' as well in the presented figure.

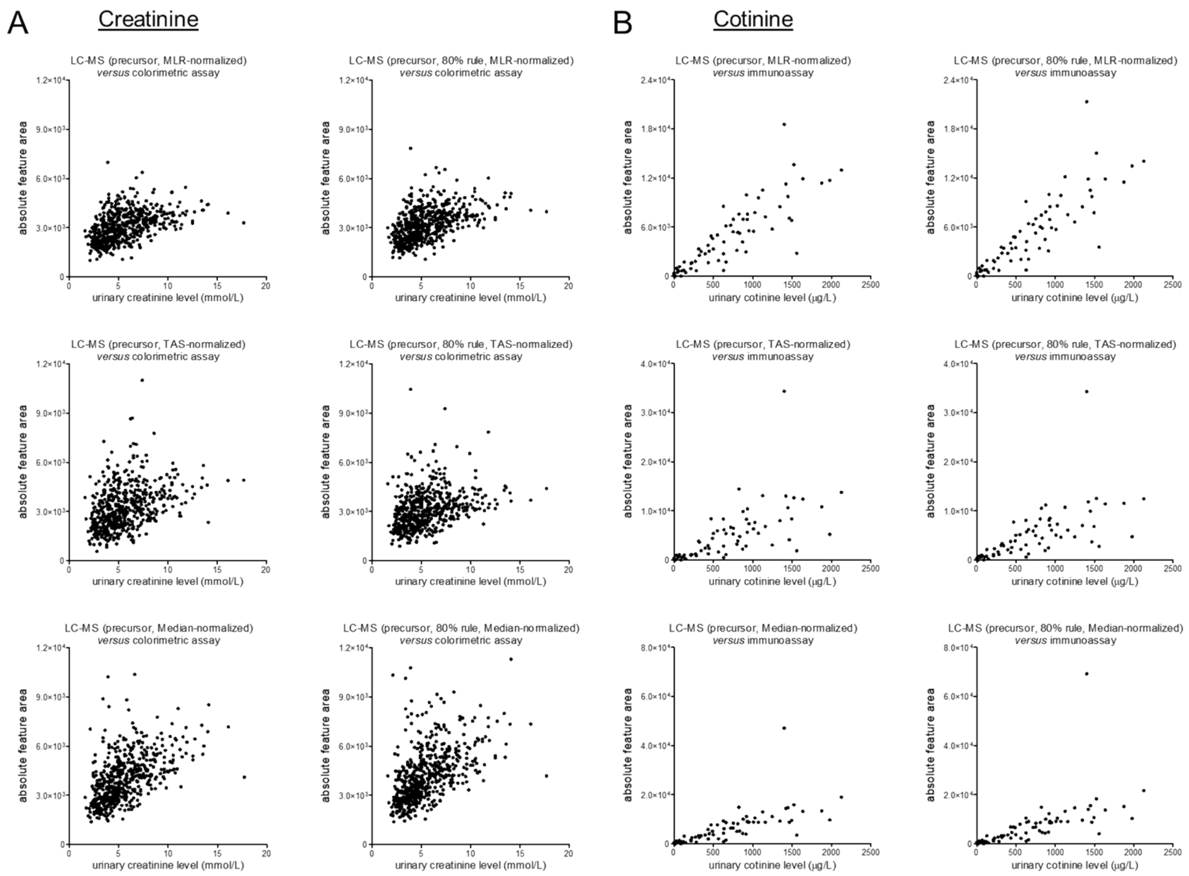

Figure S23. Scatter plots presenting MLR- (top), TAS- (middle), and Median-normalized (bottom) feature data of the MS1-level precursors on the y-axis and the results of previously-conducted routine measurements (in an ISO 15189 certified laboratory) on the x-axis for (**A**) the endogenous muscle breakdown product creatinine and (**B**) the exogenous phase I nicotine metabolite cotinine. Plots on the left represent results obtained by directly normalizing the data after feature finding whereas normalization factors of the plots on the right where obtained after first filtering data using the '80% rule' as described by Bijlsma *et al*. (Analytical Chemistry, 2006, 78, 567–574).
